# Integrative multi-omics analysis reveals the domestication mechanism of black rice

**DOI:** 10.1093/nsr/nwaf497

**Published:** 2025-11-13

**Authors:** Zaihui Zhou, Zenan Yang, Qinglu Zhang, Xushi Wang, Qiu Chen, Xinyao Yu, Yao Zhou, Yidan Ouyang, Jianwei Zhang, Weibo Xie, Qifa Zhang, Hao Chen

**Affiliations:** National Key Laboratory of Crop Genetic Improvement, Hubei Hongshan Laboratory, Huazhong Agricultural University, Wuhan 430070, China; Shenzhen Institute of Nutrition and Health, Huazhong Agricultural University, Wuhan 430070, China; Shenzhen Branch, Guangdong Laboratory for Lingnan Modern Agriculture, Genome Analysis Laboratory of the Ministry of Agriculture, Agricultural Genomics Institute at Shenzhen, Chinese Academy of Agricultural Sciences, Shenzhen 518120, China; National Key Laboratory of Crop Genetic Improvement, Hubei Hongshan Laboratory, Huazhong Agricultural University, Wuhan 430070, China; Shenzhen Institute of Nutrition and Health, Huazhong Agricultural University, Wuhan 430070, China; Shenzhen Branch, Guangdong Laboratory for Lingnan Modern Agriculture, Genome Analysis Laboratory of the Ministry of Agriculture, Agricultural Genomics Institute at Shenzhen, Chinese Academy of Agricultural Sciences, Shenzhen 518120, China; National Key Laboratory of Crop Genetic Improvement, Hubei Hongshan Laboratory, Huazhong Agricultural University, Wuhan 430070, China; Shenzhen Institute of Nutrition and Health, Huazhong Agricultural University, Wuhan 430070, China; Shenzhen Branch, Guangdong Laboratory for Lingnan Modern Agriculture, Genome Analysis Laboratory of the Ministry of Agriculture, Agricultural Genomics Institute at Shenzhen, Chinese Academy of Agricultural Sciences, Shenzhen 518120, China; National Key Laboratory of Crop Genetic Improvement, Hubei Hongshan Laboratory, Huazhong Agricultural University, Wuhan 430070, China; Shenzhen Institute of Nutrition and Health, Huazhong Agricultural University, Wuhan 430070, China; Shenzhen Branch, Guangdong Laboratory for Lingnan Modern Agriculture, Genome Analysis Laboratory of the Ministry of Agriculture, Agricultural Genomics Institute at Shenzhen, Chinese Academy of Agricultural Sciences, Shenzhen 518120, China; National Key Laboratory of Crop Genetic Improvement, Hubei Hongshan Laboratory, Huazhong Agricultural University, Wuhan 430070, China; Shenzhen Institute of Nutrition and Health, Huazhong Agricultural University, Wuhan 430070, China; Shenzhen Branch, Guangdong Laboratory for Lingnan Modern Agriculture, Genome Analysis Laboratory of the Ministry of Agriculture, Agricultural Genomics Institute at Shenzhen, Chinese Academy of Agricultural Sciences, Shenzhen 518120, China; National Key Laboratory of Crop Genetic Improvement, Hubei Hongshan Laboratory, Huazhong Agricultural University, Wuhan 430070, China; Shenzhen Institute of Nutrition and Health, Huazhong Agricultural University, Wuhan 430070, China; Shenzhen Branch, Guangdong Laboratory for Lingnan Modern Agriculture, Genome Analysis Laboratory of the Ministry of Agriculture, Agricultural Genomics Institute at Shenzhen, Chinese Academy of Agricultural Sciences, Shenzhen 518120, China; State Key Laboratory of Forage Breeding-by-Design and Utilization, Institute of Botany, Chinese Academy of Sciences, Beijing 100093, China; National Key Laboratory of Crop Genetic Improvement, Hubei Hongshan Laboratory, Huazhong Agricultural University, Wuhan 430070, China; National Key Laboratory of Crop Genetic Improvement, Hubei Hongshan Laboratory, Huazhong Agricultural University, Wuhan 430070, China; National Key Laboratory of Crop Genetic Improvement, Hubei Hongshan Laboratory, Huazhong Agricultural University, Wuhan 430070, China; National Key Laboratory of Crop Genetic Improvement, Hubei Hongshan Laboratory, Huazhong Agricultural University, Wuhan 430070, China; National Key Laboratory of Crop Genetic Improvement, Hubei Hongshan Laboratory, Huazhong Agricultural University, Wuhan 430070, China; Shenzhen Institute of Nutrition and Health, Huazhong Agricultural University, Wuhan 430070, China; Shenzhen Branch, Guangdong Laboratory for Lingnan Modern Agriculture, Genome Analysis Laboratory of the Ministry of Agriculture, Agricultural Genomics Institute at Shenzhen, Chinese Academy of Agricultural Sciences, Shenzhen 518120, China

**Keywords:** *Oryza sativa*, black rice, crop domestication, anthocyanin biosynthesis, GWAS

## Abstract

Black rice, a nutritionally superior yet genetically uncharacterized germplasm, exhibits extreme rarity (∼ 1%) in natural rice populations. Here, we establish a global germplasm collection of 367 black rice accessions and employ integrated multi-omics (genomics, transcriptomics, metabolomics) to dissect its divergence from white rice. Population genomic analyses reveal a two-step domestication trajectory for black rice from the wild progenitor: initial *Rc*-mediated loss of proanthocyanidin pigmentation, followed by *OsKala4*/*OsMYB3*-driven anthocyanin activation under artificial selection. Genome-wide scans identify 238 differentially selected regions between black and white rice that orchestrate extensive transcriptomic reprogramming (up to 22% differentially expressed genes) and metabolomic remodeling (up to 32% differentially accumulated metabolites) in black rice seeds. Multi-omics analysis and a genome-wide association study uncover previously uncharacterized regulators (*OsWRKY1, OsBBX13, OsTT12*) that synergistically enhance the MYB-bHLH-WD40 (MBW) transcriptional complex to regulate anthocyanin biosynthesis and vacuolar sequestration. Furthermore, we reconstructed a comprehensive biosynthetic pathway network for phenolic acids and flavonoids in black rice through integration of experimental metabolomic and transcriptomic datasets with Kyoto Encyclopedia of Genes and Genomes (KEGG) pathway annotations. Finally, we evaluated the selection status of 225 quantitative trait genes and compared agronomic traits between black and white rice. Our findings provide a molecular roadmap for precision breeding strategies to develop elite black rice cultivars with optimized nutritional and agronomic performance.

## INTRODUCTION

As a staple food for over half the world’s population, rice (*Oryza sativa*) contributes approximately 20% of global calorie intake, making it a critical crop for food security [1]. As a research model for monocot plants, rice possesses extensive germplasm resources characterized by remarkable phenotypic and genetic diversity. This diversity not only serves as an invaluable foundation for investigating crop evolutionary trajectories but also provides essential genetic material for breeding strategies aimed at enhancing agronomic and adaptive traits. The advance of next-generation sequencing has revolutionized multi-omics research, empowering correlative analysis of genomic, transcriptomic and metabolomic datasets to decode the molecular foundation of complex traits [2]. Over the past two decades, multi-omics approaches including genomics, transcriptomics and metabolomics have been widely applied to dissect rice germplasm populations, uncovering genetic mechanisms underlying its domestication, environmental adaptation and key agronomic traits [3–12]. These discoveries have not only significantly deepened our understanding of rice evolutionary dynamics but also accelerate the development of precision breeding programs targeting yield improvement and environment resilience.

Rice germplasm exhibits natural variation in pericarp coloration, ranging from white to red and black/purple. Although most modern rice cultivars exhibit white pericarp, their wild progenitor *Oryza rufipogon* typically displays red pericarp. The white rice phenotype originates from loss-of-function mutations in the *OsRc* gene, which abolishes proanthocyanidin biosynthesis [13], while the formation of black pericarp arises from structural rearrangements within the promoter region of a bHLH transcription factor gene *OsKala4*, which activates *OsKala4* transcription thereby driving anthocyanin biosynthesis in the pericarp tissue [14]. Analyses of major rice diversity panels—including the 3000 Rice Genomes Project population and a globally representative core collection of 533 accessions—reveal a striking imbalance in pigmentation prevalence; over 85% of accessions display white pericarp, approximately 10%–14% accumulate proanthocyanidins responsible for red pigmentation, and about 1% produce anthocyanins that confer black or purple hues [7,15].

The pigments in rice pericarp not only impart distinctive visual traits but also elevate nutritional quality by enhancing bioactive compound contents [16,17]. Comprehensive nutrient analyses reveal that whole-grain rice retains significantly higher levels of fiber, essential minerals, vitamins and bioactive compounds compared with milled rice, with black rice varieties demonstrating exceptional enrichment in certain phytochemicals including anthocyanins, proanthocyanidins, phenolic acids and carotenoids etc [18]. The nutritional superiority of whole-grain black rice is quantifiable through its Nutrient Density Unit (NDU = 1.1098) surpassing milled white rice (NDU = 0.4356) and whole-grain white rice (NDU = 0.9522) by 154.8% and 16.6%, respectively [18]. Substantial evidence from animal studies validates the health benefits of black rice consumption, including antioxidant capacity, anti-inflammatory effects, prevention of chronic non-communicable diseases (e.g. type 2 diabetes, cardiovascular disorders and certain cancers) and lifespan extension [16]. Recognizing these advantages, the scientific community has begun to advocate for adopting whole-grain black rice as a primary dietary staple in rice-consuming communities [19]. Furthermore, a novel breeding initiative termed Green Nutritious Super Rice (GNSR) has been proposed to synergistically address food security, agricultural sustainability and public health. This strategy employs whole-grain black rice as a prototype for developing nutrient-dense cultivars [20].

Despite the widespread application of multi-omics approaches in rice germplasm research, the genomic, transcriptomic and metabolomic profiles of black rice accessions remain largely uncharacterized. This knowledge gap stems from their scarcity in natural rice populations, which critically hinders the development of elite black rice cultivars and their commercial promotion. To address this limitation, in this study, we collected a worldwide population comprising 367 black rice accessions. Utilizing this black rice resource, we conducted a pioneering investigation into the evolutionary origin and domestication history of black rice varieties through genomic and phylogenetic analyses. Furthermore, systematic multi-omics profiling elucidated the genetic regulatory mechanism and metabolic pathways underlying anthocyanin biosynthesis—the hallmark biochemical distinction between black and white rice varieties.

## RESULTS

### Collection and sequencing of a black rice population

A total of 367 black rice accessions, including landraces and cultivars, were collected from China, Indonesia, Japan, Myanmar and the Philippines (Table S1). These accessions show substantial natural variation in anthocyanin content of seeds, ranging from 7.14 to 8434.83 µg/g dry weight (Fig. S1a and b). Genomes of all accessions were sequenced on with an average depth of ∼20× (ranging from 13.8× to 37.0×). To identify adaptive genomic patterns in black rice, we supplemented these data with publicly available genomes from 185 wild rice (*O. rufipogon*) accessions [21] and 202 high-coverage (>30×) white rice accessions from the 3000 Rice Genomes Project [5,7] (Table S2). We devised a high-throughput analytical pipeline integrating genomic, transcriptomic and metabolomic datasets to systematically interrogate multi-omics data (Fig. S2).

**Figure 1.**
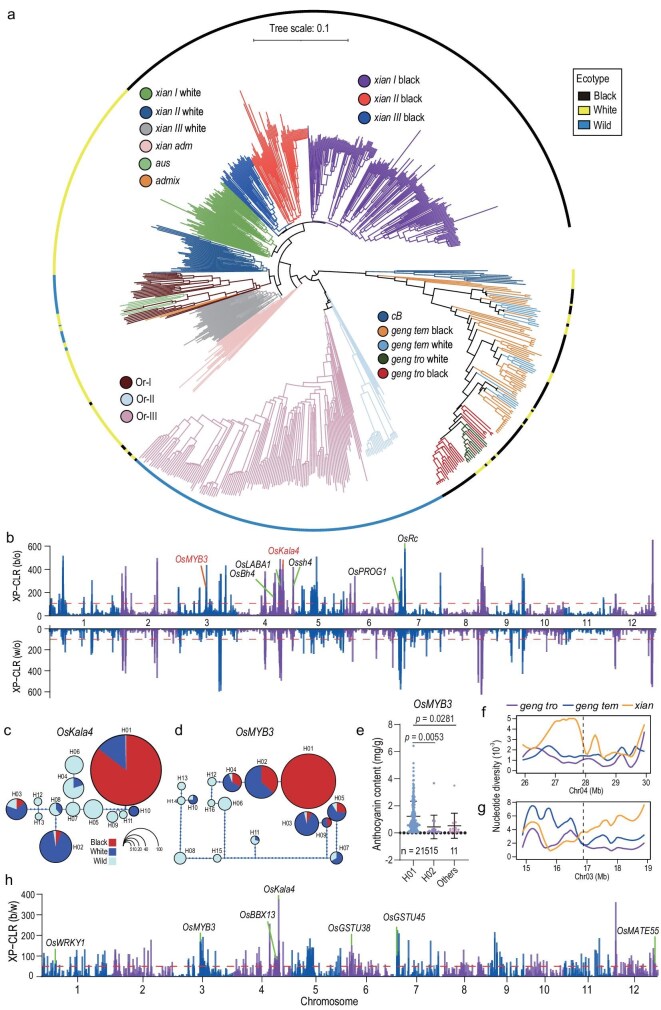
Screening and identification of selective sweep loci in black rice. (a) Neighbor-joining phylogenetic tree of all 754 rice accessions. (b) Genome-wide XP-CLR analysis between black rice/*O. rufipogon* (top) and white rice/*O. rufipogon* (bottom) using 100-kb sliding windows with 10-kb steps. (c and d) Haplotype network of *OsKala4* (c) and *OsMYB3* (d). Circle sizes in (c) and (d) represent haplotype frequencies with those below 0.5% excluded. (e) Anthocyanin content variation among *OsMYB3* haplotypes. *P* values were determined by the two-tailed Student’s *t*-test. (f and g) Nucleotide diversity (π) distribution within 4-Mb genomic regions surrounding *OsKala4* (f) and *OsMYB3* (g). Gene loci are indicated by black dashed lines. (h) Selective sweep detection between black and white rice using XP-CLR (100-kb sliding windows with 10-kb steps).

Clean paired-end reads from all 754 rice genomes were individually mapped to the Nipponbare reference genome (IRGSP-1.0), identifying approximately 4.3 million single nucleotide polymorphisms (SNPs). A neighbor-joining phylogenetic tree constructed using high-quality synonymous SNPs revealed clear divergence of subspecies from wild groups (Fig. 1a and Fig. S3a–S3c). The analysis delineated two distinct groups corresponding to *geng* (*japonica*) and *xian* (*indica*) black rice, comprising 91 and 276 accessions, respectively (Fig. S3a). The *geng* black rice formed two subgroups (*temperate* and *tropical Geng*), while the *xian* black rice comprised three subgroups (*xian I, xian II* and *xian III*) plus a *xian* admixture. *Xian* black rice clustered in branches distinct from *xian* white rice (Fig. 1a). Although some admixing occurred between *Geng* black and white rice, their primary branches predominantly contained single rice types (Fig. 1a). Principal component analysis (PCA) clearly separated the black rice population into *xian* and *geng* groups (Fig. S1c). Population structure analysis using the ADMIXTURE program with increasing *K*-values (2–6) further validated these genetic divisions (Fig. S1d).

### Domestication loci of black rice

To investigate the domestication process of black rice, we analyzed selective signatures between black rice and *O. rufipogon* (B–O) using the cross-population composite likelihood ratio (XP-CLR) method (Fig. 1b, Table S3). For comparison, we similarly calculated XP-CLR scores between white rice and *O. rufipogon* (W–O) (Fig. 1b, Table S4). We defined selective sweeps as genomic regions within the top 1% of XP-CLR scores, identifying 103 domestication/selective sweep loci containing 2180 non-transposable element (non-TE)-associated genes across all black rice accessions (Fig. 1b and Table S3). Approximately 50.1% of these domestication/selective sweep loci overlapped with those detected in white rice. Subgroup analyses identified 165 loci (3149 non-TE genes) in *geng* black rice and 178 loci (3016 non-TE genes) in *xian* black rice (Fig. S4). Key domestication genes within these loci included *OsPROG1* (plant architecture) [22–24], *OsRc* (pericarp color) [13], *OsBh4* (*Blackhull 4*, hull colors) [25], *OsLABA1* (awn length and loss of barbs) [26] and *Ossh4* (seed shattering) [27–29]. These genes were shared between black and white rice domestication loci (Fig. 1b). Haplotype analyses further confirmed that identical superior haplotypes of these genes were maintained in both black and white rice populations (Fig. S5a–S5e), demonstrating their shared early domestication trajectories from a common wild ancestor.

However, we identified two genomic regions containing anthocyanin regulatory genes, *OsKala4* [14] and *OsMYB3* [30], which exhibited exclusive selection signals in black rice compared to white rice (Fig. 1b). Both *OsKala4* and *OsMYB3* showed signatures of strong positive selection, as evidenced by their elevated population differentiation (*F*_st_) of B–O compared to W–O and reduced nucleotide diversity (π) value compared to *O. rufipogon* and white rice (Fig. S6a–S6d).

*OsKala4*, a key gene associated with black rice pigmentation, acquired its function through an 11-kb insertion upstream of its transcription start site, uniquely conserved in black rice [14, 31]. Our previous study localized this functional rearrangement to the −3 kb to −4 kb upstream region [31]. We performed haplotype analysis of the 11-kb promoter region across our black rice population. This revealed an exceptionally conserved sequence with only a single SNP, defining two haplotypes: the predominant H01 (98.9% of varieties); and a rare H02 (found in only four accessions) (Fig. S7). This pattern strongly indicates a single evolutionary origin for the functional *OsKala4* allele. Although no coding sequence variations in *OsKala4* were detected between black and white rice, haplotype analysis spanning its 5′-UTR to 3′-UTR demonstrated that 95.9% of black rice accessions clustered into haplotype H01 (Fig. 1c), establishing a robust association between this haplotype and the black rice phenotype.

*OsMYB3* encodes another core anthocyanin regulatory gene in black rice [30]. Haplotype analysis identified a black rice-specific haplotype (H01) present in 79.3% of black rice accessions, but completely absent in white and wild rice (Fig. 1d). Notably, accessions carrying *OsMYB3*-H01 exhibited significantly higher average seed anthocyanin content compared to other haplotypes (Fig. 1e), indicating that H01 represents a functionally enhanced allele selected during black rice improvement to enhance pericarp pigmentation.

*Tropical geng* black rice exhibited significantly lower nucleotide diversity (π = 0.0013) in the *OsKala4* locus compared to *temperate Geng* (π = 0.0016) and *xian* black rice (π = 0.0028) (Fig. 1f). This result suggests that functional *OsKala4* likely originated in *tropical geng* populations before spreading to other subgroups through introgression—a hypothesis consistent with a 2015 study by Oikawa *et al.* [14]. Unlike *OsKala4*, which displayed divergent π values between *temperate* and *tropical geng* subgroups, *OsMYB3* maintained uniformly low π values across *geng* subpopulations (Fig. 1g). This pattern implies that *OsMYB3* was initially fixed in ancestral *geng* black rice prior to its introgression into *xian* subgroups.

It is notable that the *OsRc* gene responsible for red pericarp pigmentation showed similar selection patterns in black and white rice, with only 5.4% of black rice accessions (20 out of 367) retaining functional *OsRc* alleles (Fig. S5e). Direct selection for non-functional *rc* alleles in black rice through pericarp color is challenging, as black pigmentation may mask red coloration. This observation supports a sequential domestication model in which black rice emerged after the establishment of white rice, rather than directly from wild progenitors.

### Differential selection signatures in genomic regions of black vs. white rice

To investigate genetic differentiation between black and white rice, we identified differentially selected regions in black rice in contrast to white rice using the XP-CLR method. Genome-wide scans employed 100-kb sliding windows with regions in the top 1% of XP-CLR scores defined as differentially selected. This analysis revealed 238 candidate regions containing 2656 non-TE-associated genes, representing 7.0% of annotated genes in the reference genome (Fig. 1h, Table S5).

To assess potential false positives among the 238 candidate selected regions arising from genetic linkage with *OsKala4*, we performed a targeted linkage disequilibrium (LD) analysis of the genomic region surrounding *OsKala4*. We selected SNPs within a 1-Mb window centered on *OsKala4* and computed pairwise LD (*r*^2^). The results indicate no strong LD between the *OsKala4* locus and adjacent regions; notably, the selection interval containing *OsKala4* (27.78–28.21 Mb) showed no significant LD with the nearest XP-CLR region (26.84–27.08 Mb) (Fig. S8), ruling out substantial physical linkage. Given the overall lack of high LD around *OsKala4*, very few—if any—of the 238 signals are artifactual due to linkage.

Kyoto Encyclopedia of Genes and Genomes (KEGG) pathway analysis of these 2656 non-TE-associated genes within the selected region identified eight significantly enriched pathways, including biosynthesis of various plant secondary metabolites (Fig. S9). Because the most prominent difference between black and white rice is their pericarp color, we infer that these differentially selected regions are related to enhancement of the biosynthesis of anthocyanin and other pigmented secondary metabolites, although many unrelated genes therefore hitchhike during this selective process. Besides the black rice-specific *OsKala4* and *OsMYB3*, the differentially selected genes also included other transcriptional factor genes from MYB, bHLH, WRKY, bZIP and BBX families. Members of these transcription factor families have been reported to be involved in regulating anthocyanin biosynthesis in plants. Additionally, five transport genes (*OsGSTU6, OsGSTU38, OsGSTU45, OsMATE31* and *OsMATE55*) were also identified within the differentially selected regions. Members of these families are known to mediate anthocyanin or flavonoid transport from the cytoplasm into vacuoles for storage after biosynthesis [32]. Notably, no catalytic enzyme genes directly involved in anthocyanin biosynthesis were detected among the differentially selected genes.

### Transcriptomic and metabolomic differences between black and white rice

**Figure 2.**
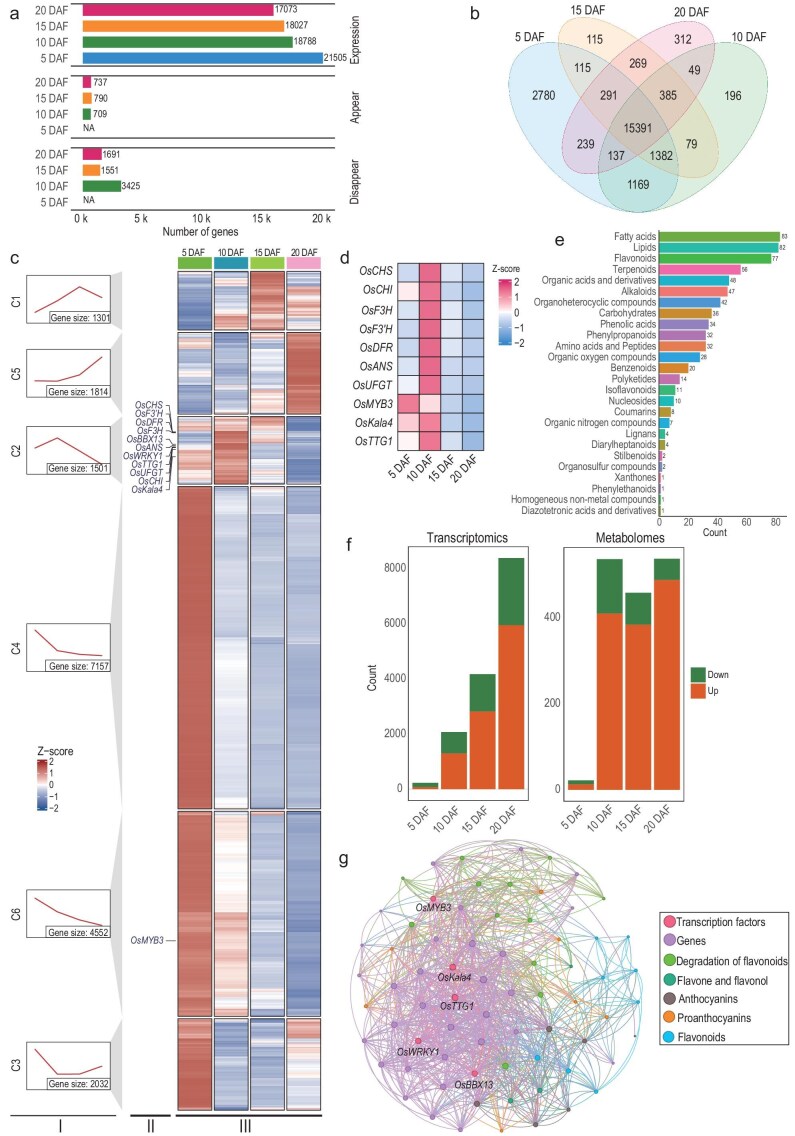
Transcriptomic and metabolomic dynamics during black rice seed development. (a) Global transcriptome profiles across developmental stages. (b) Venn diagram of stage-specific expressed genes. (c) K-means clustering (*k* = 6) of developmentally regulated genes: I. Cluster-specific expression trajectories; II. Enrichment of anthocyanin-related gene within clusters; III. Heatmap visualization of cluster expression patterns. (d) Expression dynamics of anthocyanin biosynthesis genes during development. (e) Classification of all differentially accumulated metabolites. (f) Number of differentially expressed genes (left) and differentially accumulated metabolites (right) identified at different seed developmental stages. (g) Correlation-based co-expression network of key anthocyanin-related metabolites and genes.

Using transcriptomic and metabolomic analyses, we systematically profiled the temporal dynamics of gene expression and metabolite accumulation in developing seeds of black rice, and further compared these patterns with those of white rice. We first performed RNA-Seq using developing seeds from 18 representative black rice accessions and 20 white rice accessions at four developmental stages, i.e. 5 days after flower (DAF), 10 DAF, 15 DAF and 20 DAF. About 60.5% (22 909/37 838) of the annotated genes of the reference genome were expressed during the seed development of black rice, and about 40.7% (15 391/37 838) were constantly expressed throughout all the four developmental stages (Fig. 2a and b).

Hierarchical clustering of transcriptome data across developmental stages delineated six distinct expression patterns (Clusters 1–6), each representing genes with coordinated transcriptional dynamics (Fig. 2c). We further analyzed the expression patterns of all known anthocyanin biosynthetic and regulatory genes. The results revealed that all anthocyanin biosynthetic genes (*OsCHS, OsCHI, OsF3′H, OsF3H, OsDFR, OsANS* and *OsUFGT*) and regulatory genes (*OsKala4* and *OsTTG1*) were clustered in Cluster 2, except for *OsMYB3*. These genes exhibited a coordinated expression pattern characterized by an initial upregulation during the early seed developmental stage (5–10 DAF), peaking at 10 DAF, followed by a gradual downregulation in later developmental phases (Fig. 2c and d), while *OsMYB3* was assigned to Cluster 6, a group characterized by transcriptional dynamics involving an early sharp expression peak (5 DAF) followed by progressive attenuation during seed development (Fig. 2c and d).

To systematically map the developmental metabolome divergence between black rice and white rice, we performed non-targeted metabolomic profiling of developing seed samples collected from 18 representative black rice accessions and 20 white rice accessions at four distinct developmental stages (5, 10, 15 and 20 DAF). We identified a total of 17 890 analytes in developing seeds of both black rice and white rice, and of these, 1675 metabolites were annotated with high confidence (Table S6). These annotated metabolites mainly belonged to flavonoids (including anthocyanins, proanthocyanin, flavones, flavans), fatty acids, lipids, terpenoids, phenolic acids, amino acids, organic acids (including certain vitamins), alkaloids (including certain vitamins) and others (Fig. 2e).

We compared transcriptome and metabolome datasets from developing seeds of black and white rice. This analysis revealed 227–8386 differentially expressed genes (DEGs) and 21–537 differentially accumulated metabolites (DAMs) in black rice (Fig. 2f). Notably, black rice exhibited a significant predominance of upregulated DEGs and DAMs over downregulated counterparts. Comparative profiling at 5 DAF detected minimal molecular divergence between the black and white rice despite peak transcript abundance at this stage, indicating negligible transcriptomic and metabolomic differentiation during early seed development prior to activation of core anthocyanin biosynthesis regulators and their enzymatic cascades.

Synchronized activation of most DEGs and DAMs initiated at 10 DAF (Fig. 2f) temporally aligned with anthocyanin pathway induction. These coordinated molecular patterns underlie the metabolic signature of black rice: tissue-specific hyperaccumulation of flavonoids—particularly anthocyanins—in the pericarp, a trait absent in white rice.

We subsequently constructed a co-expression network to elucidate interactions among anthocyanin-related genes, integrating selective sweep loci with transcriptomic and metabolomic datasets (Fig. 2g). This network establishes a framework for pinpointing regulatory genes critical to anthocyanin biosynthesis in black rice. Beyond the previously reported components of the MYB-bHLH-WD40 (MBW) complex (i.e., *OsMYB3, OsKala4* and *OsTTG1*) [14,30,33], our analysis revealed that other transcription factors such as *OsWRKY1* and *OsBBX13* exhibited strongly positive correlations with both flavonoid metabolite accumulation and the expression of anthocyanin biosynthetic genes.

### Identification of regulatory genes of anthocyanin biosynthesis in black rice via multi-omics analysis

**Figure 3.**
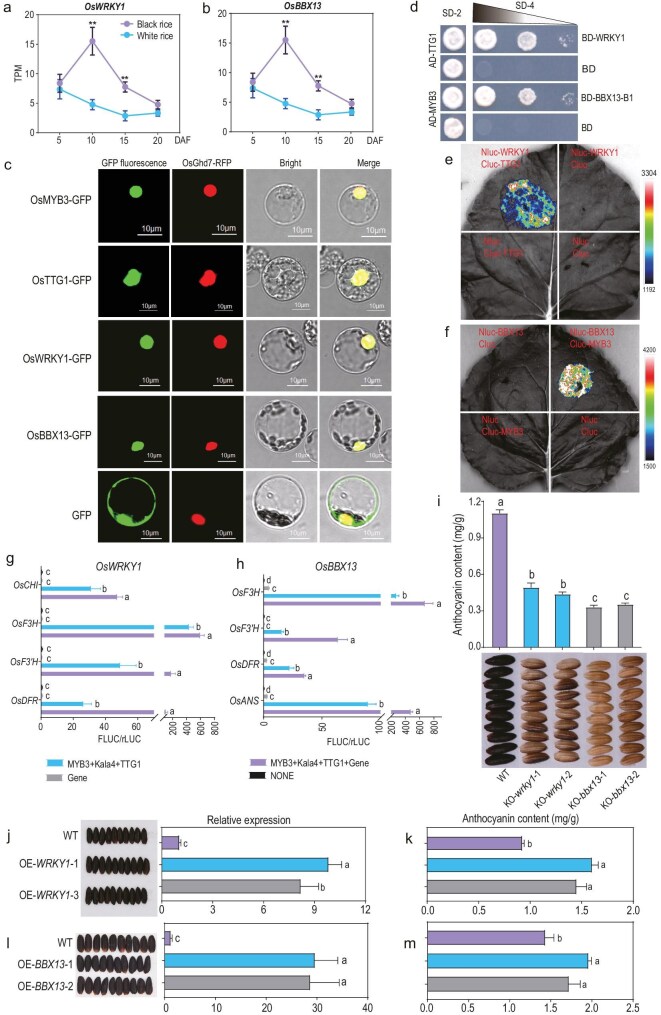
Functional validation of *OsWRKY1* and *OsBBX13*. (a and b) Expression levels of *OsWRKY1* (a) and *OsBBX13* (b). ** indicates statistical significance at *P* < 0.01 according to the two-tailed Student’s *t*-test. (c) Subcellular localization of OsBBX13, OsWRKY1, OsMYB3 and OsTTG1. (d) Yeast-two-hybrid validating the direct interaction of OsWRKY1 with OsTTG1, and OsBBX13 with OsMYB3. (e and f) LCI assay in *Nicotiana benthamiana* leaves confirming direct interaction of OsWRKY1 with OsTTG1 (e) and OsBBX13 with OsMYB3 (f). (g and h) Dual-luciferase assays showing transcriptional activation of anthocyanin biosynthetic genes by OsWRKY1 (g) and OsBBX13 (h). (i) Phenotypic comparison and anthocyanin quantification in *OsWRKY1* and *OsBBX13* knockout lines (*n* = 3). (j) Analysis of *OsWRKY1* overexpression lines: phenotypes of mature seeds and target gene expression in developing seeds at 10 DAF (*n* = 3). (k) Anthocyanin content in mature seeds of *OsWRKY1* overexpression lines (*n* = 3). (l) Analysis of *OsBBX13* overexpression lines: phenotypes of mature seeds and target gene expression in developing seeds at 10 DAF (*n* = 3). (m) Anthocyanin content in seeds of *OsBBX13* overexpression lines. All data indicate mean ± SD. Different lowercase letters indicate significant differences; *P* < 0.05, one-way analysis of variance (ANOVA) with Tukey’s honestly significant difference (HSD) test.

Through integrated genomic, transcriptomic and metabolomic analyses, we identified previously uncharacterized regulatory genes controlling anthocyanin biosynthesis in black rice, specifically *OsWRKY1* and *OsBBX13*. The two transcription factor genes are not only located within genomic regions under differential selection between black and white rice (Fig. 1h), but also serve as central hubs within the co-expression network of anthocyanin pathway genes (Fig. 2g). Furthermore, *OsWRKY1* and *OsBBX13* exhibit marked differential expression during seed development between black and white rice (Fig. 3a and b). We further confirm their functions in modeling anthocyanin accumulation. Subcellular localization analysis demonstrated that OsBBX13, OsWRKY1, OsTTG1 and OsMYB3 were localized to the nucleus (Fig. 3c), consistent with their functional roles as transcriptional regulators. Protein–protein interaction assays, including yeast-two-hybrid and luciferase complementation imaging (LCI), further confirmed that OsWRKY1 interacts with OsTTG1, and OsBBX13 interacts with OsMYB3 (Fig. 3d–f). The dual-luciferase assay revealed that OsWRKY1 or OsBBX13 alone exhibited negligible or minimal activation effects on anthocyanin biosynthetic gene expression (Fig. 3g and h). However, in the presence of the MBW complex (OsMYB3-OsKala4-OsTTG1), OsWRKY1 specifically amplified the MBW complex-mediated activation of *OsF3′H* and *OsDFR*, while OsBBX13 synergistically enhanced the MBW complex-mediated transcriptional activation of *OsF3H, OsF3′H* and *OsANS* (Fig. 3g and h).

Knockout of *OsWRKY1* or *OsBBX13* in the black rice accession W110 caused significantly attenuated seed anthocyanin content to 29.8%–44.5% of the WT control (Fig. 3i and Fig. S10). In contrast, overexpression of *OsWRKY1* and *OsBBX13* in W110 resulted in a significant increase in seed anthocyanin content by 57.4%–73.6% and 20.2%–36.8%, respectively (Fig. 3j–m). These results suggest that anthocyanin biosynthesis is the limiting factor for anthocyanin accumulation in this black rice background, and that both genes function as positive regulators that enhance the expression of upstream biosynthetic genes.

### Genome-wide association study (GWAS) for anthocyanin content in black rice

We further conducted a GWAS for the black pericarp trait and seed anthocyanin content. First, we performed a GWAS for pericarp color across a mixed population comprising 367 black rice accessions and 202 white rice accessions. The results showed that the *OsKala4* locus exhibited the strongest association signal, while *OsMYB3* and *OsWRKY1* were located near additional peak regions (Fig. S11).

**Figure 4.**
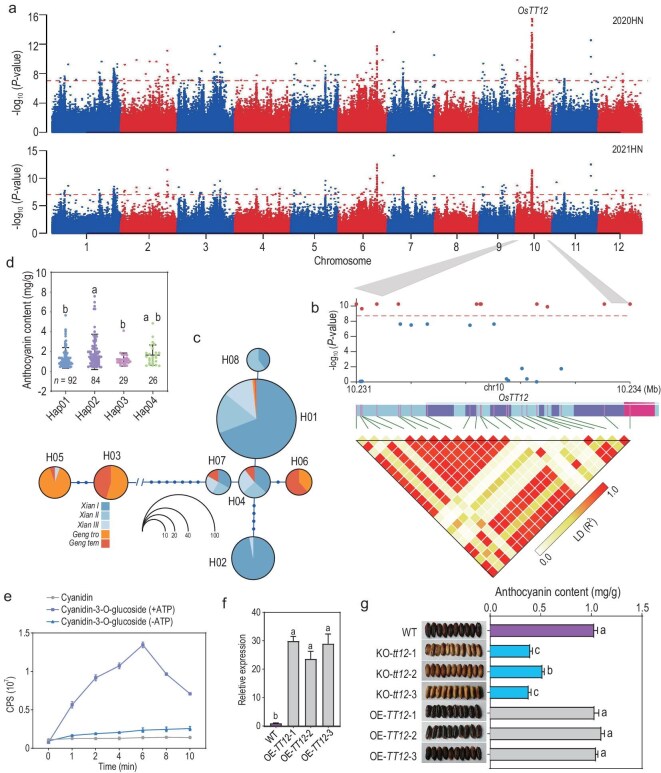
GWAS for seed anthocyanin content using the black rice population. (a) Manhattan plot for seed anthocyanin content in 2020 and 2021. (b) Distribution of SNPs in *OsTT12*. (c) Haplotype network of *OsTT12*. (d) Anthocyanin content of different haplotypes. (e) Validation of the transport capability of OsTT12 for cyanidin-3-*O*-glucoside in yeast microsomes. CPS, count per second. (f) Target gene expression in developing seeds of *OsTT12* overexpression at 10 DAF. (g) Phenotypes and anthocyanin content of mature seeds of *OsTT12* knockout and overexpression lines. All data indicate mean ± SD. Different lowercase letters indicate significant differences; *P* < 0.05, one-way ANOVA with Tukey’s HSD test.

Since the *OsKala4* gene plays a decisive role in black rice formation, its strong effect may mask or interfere with the detection of other loci contributing to pericarp coloration. To minimize this confounding effect, we subsequently performed a GWAS specifically for seed anthocyanin content using only the black rice population over two consecutive years (2020–21) (Fig. 4a). This analysis identified 309 significantly associated loci, with a consistently significant locus at 10.08 Mb on chromosome 10, showing interannual reproducibility (Fig. 4b). Within this locus, we identified a rice gene ortholog (designated *OsTT12*) of the *Arabidopsis thaliana* multidrug and toxic compound extrusion (MATE) transporter TRANSPARENT TESTA12 (TT12), which mediates the transport of cyanidin-3-*O*-glucoside [34]. Haplotype analysis revealed significant anthocyanin content variation among *OsTT12* haplotypes (Fig. 4c and d). Transport activity validation demonstrated that yeast vesicles expressing *OsTT12* transported cyanidin-3-*O*-glucoside but not cyanidin (Fig. 4e), similar to *Arabidopsis* TT12 activity [34].

*OsTT12* knockout lines exhibited a partial but significant reduction in anthocyanin content, reaching 36.7%–50.0% compared to the W110 wild-type control, suggesting functional redundancy in anthocyanin transport. However, overexpression of *OsTT12* in W110 did not significantly alter seed anthocyanin levels (Fig. 4f and g). The possible explanation is that downstream transport is not the bottleneck for anthocyanin accumulation in W110—in contrast to *OsWRKY1* and *OsBBX13* overexpression in W110, which confirmed that upstream anthocyanin biosynthesis is the limiting factor for anthocyanin accumulation in this background. These findings confirm OsTT12 as a functional transporter contributing to anthocyanin accumulation while implicating the existence of redundant transporters in black rice pigmentation.

### Integrated biosynthetic pathway of phenolic acid and flavonoid in black rice

The health-promoting properties of black rice are largely attributed to its enriched polyphenolic profile including anthocyanins, proanthocyanidins and phenolic acids compared to white rice. These bioactive compounds are biosynthesized through the phenylpropanoid pathway. Our non-targeted metabolomic analysis identified a wide range of phenolic acid and flavonoid metabolites (Table S6). Among these, several delphinidin-derived anthocyanins, such as delphinidin 3-*O*-glucoside and malvidin 3-*O*-glucoside, were tentatively detected. Although it has conventionally been held that black rice lacks delphinidin-derived anthocyanins, our non-targeted results are consistent with several recent studies, including the review by Chen *et al.* [35], which reported the presence of such pigments in black rice.

Given that non-targeted metabolomic identifications can be unreliable due to challenges in distinguishing isobaric and isomeric compounds, incomplete reference spectral libraries and the probabilistic nature of matching algorithms, we further performed targeted metabolite analysis to validate these findings. We focused on anthocyanin profiling using whole-grain and bran samples from 30 distinct black rice accessions. Blue wheat, known to accumulate delphinidin-derived anthocyanins, was used as a positive control, while whole-grain red and white rice, expected to lack anthocyanins, served as negative controls (Fig. S12).

Using authentic standards, we confirmed the presence of seven major anthocyanin species. Their average concentrations, in descending order, were as follows: cyanidin 3-*O*-glucoside > peonidin 3-*O*-glucoside > cyanidin 3-*O*-rutinoside ≈ cyanidin 3,5-di-*O*-glucoside > peonidin 3,5-di-*O*-glucoside > delphinidin 3-*O*-glucoside > malvidin 3-*O*-glucoside (Fig. S12, Table S7). The targeted analysis confirmed the detectable presence—though at low absolute concentrations—of at least two delphinidin-derived anthocyanins, delphinidin 3-*O*-glucoside and malvidin 3-*O*-glucoside, verified against authentic standards. Additionally, three other anthocyanins (peonidin 3-*O*-rutinoside, pelargonidin 3-*O*-glucoside and petunidin 3-*O*-glucoside) were identified based on published ion-pair information (Table S8).

**Figure 5.**
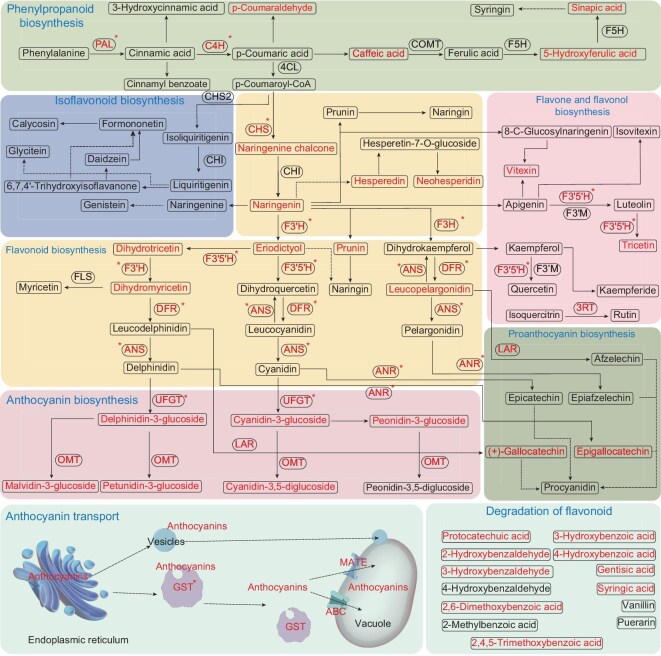
Integrated flavonoid and phenolic acid metabolic pathway in black rice. Genes or metabolites in red represent DEGs or DAMs between black and white rice. Asterisk-labeled synthases indicate enzymes encoded by genes that show divergent π between black and white rice.

By integration of our experimentally obtained metabolomic and transcriptomic datasets with KEGG pathway annotations, we reconstructed a comprehensive biosynthetic pathway network for phenolic acids and flavonoids in black rice (Fig. 5). Based on KEGG annotations, 13 metabolites detected in our non-targeted metabolomic analysis were degradation products derived from the flavonoid pathway catabolism.

We identified biosynthetic genes within this network through KEGG annotations and further analyzed their evolutionary divergence (π) and transcriptional activity (transcripts per million, TPM). This integrated approach revealed 31 genes associated with phenolic acid and flavonoid metabolism. Transcriptomic profiling identified 13 significantly upregulated genes in black rice: *OsPAL, OsPAL7, OsC4H, OsC4H1, OsCHS1, OsF3′H, OsF3H1, OsDFR, OsANS1, OsANS2, OsUFGT, OsF3′5′H1* and *OsF3′5′H2*. Subsequent population genetic analysis demonstrated significant π value differences between black and white rice accessions for *OsPAL, OsC4H, OsCHS1, OsF3′H, OsDFR, OsANS1, OsANS2, OsUFGT* and *OsF3′5′H*. Notably, anthocyanin transporters, including glutathione S-transferase (GST), MATE and ATP-binding cassette (ABC) families, exhibited differential expression patterns and selection signatures across rice cultivars. Further functional characterization of these transporters is required to elucidate their regulatory mechanisms in anthocyanin accumulation.

### Breeding status and agronomic trait evaluation of black rice

**Figure 6.**
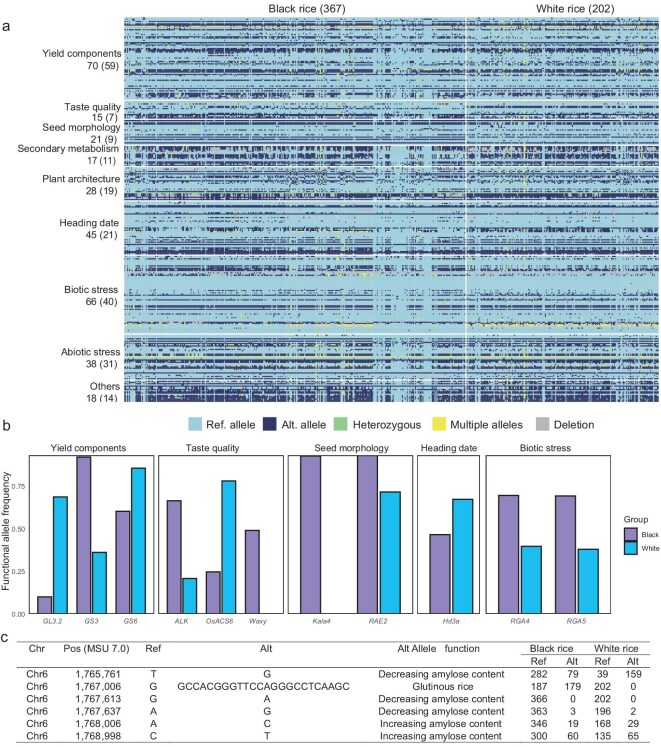
Genotype matrix of 225 QTGs in black rice and white rice accessions. (a) A total of 348 causative QTNs for 225 QTGs were genotyped for 367 black rice accessions and 202 white rice accessions. QTGs are classified into nine categories. The number of QTNs and QTGs (in brackets) for each category are shown on the left. Light blue, dark blue, light green, yellow and gray boxes represent the genotype for the Nipponbare reference (Ref.) allele, alternative (Alt.) allele, heterozygous, multiple alleles and deletion, respectively. (b) QTNs with highly differentiated allele frequency (difference > 0.2) between black and white rice subpopulations, and with an allele frequency of >0.4 in at least one subpopulation. (c) Genomic location and allelic distribution of causative QTNs in the *Waxy* gene.

Anthocyanin biosynthesis in black rice is a potential factor affecting its agronomic traits, particularly yield. Black rice varieties are generally considered to have a yield disadvantage relative to commonly cultivated white rice varieties [36]. To assess the overall breeding status of black rice, we first assessed the selection status of 225 quantitative trait genes (QTGs) in our black rice collection compared to 202 white rice accessions using RiceNavi [37]. The analysis revealed that the black rice population harbors extensive natural variation, indicating that this germplasm resource possesses substantial allelic diversity (Fig. 6a). Comparison of quantitative trait nucleotide (QTN) allele frequencies between black and white rice populations showed that most of them were similar (Table S9).

However, we also found some QTNs with relatively high allele differences between black rice and white rice subpopulations. For instance, besides the well-documented pigmentation regulator *OsKala4* (which harbors a functional QTN exclusive to black rice), we found a causative QTN conferring glutinous grain characteristics in the *Waxy* gene—a key regulator of amylose content. This QTN is present in approximately 50% of the black rice accessions but absent in all 202 white rice accessions (Fig. 6b and c). The selection preference for glutinous texture in black rice may be explained by its typical consumption as whole-grain rice. Since anthocyanins are concentrated in the pericarp, removing the bran (as in white rice) would eliminate the nutritional benefit. Therefore, the soft and palatable texture resulting from the glutinous trait after cooking was likely a key factor aligning with consumer preferences for whole-grain products.

To directly evaluate if anthocyanin biosynthesis reduces yield, we developed two pairs of black and white rice sister lines (F_6_) from two independent crosses: Huanghuazhan (white rice)/Jinzi No.1 (black rice) and Huamoxiang (black rice)/Jiayouguojing (white rice) (Fig. S13a). Each pair shared a highly similar genetic background (>96% similarity) but differed in pericarp color (black vs. white) due to the presence/absence of the functional *OsKala4* allele (Fig. S13b and S13c).

Phenotypic comparison revealed that the black rice lines consistently showed reduced yield per plant. However, the extent of reduction differed: one pair showed a statistically significant decrease of 20.7% (*P* = 0.0032), while the other showed a non-significant trend of 6.0% (*P* = 0.1103) (Fig. S13d). The yield gap was associated with significantly lower grain width, 1000-grain weight and seed-setting rate in the black lines (Fig. S13d). Notably, the pair with higher genetic similarity (98.36%) exhibited a smaller yield difference than the pair with lower similarity (96.02%) (Fig. S13b and S13c).

## DISCUSSION

Plant domestication is an artificial selection process through which humans genetically and morphologically reshape wild progenitors to meet agricultural needs. This artificial selection paradigm has enabled the development of crops with enhanced yield, edibility, storage capacity and agronomic adaptability [38–41]. Rice, domesticated from its wild ancestor *O. rufipogon* approximately 13 000 years ago, exemplifies this transformative process [42–44]. Through sustained selection for favorable alleles, early farmers shaped critical domestication syndromes including reduced seed shattering (*Ossh4*) [27–29], reduced awn length and loss of barbs (*OsLABA1*) [26], optimized plant architecture (*OsPROG1*) [22–24], reduced hull color (*OsBh4*) [25] etc. Crucially, archaeogenetic evidence supports a single-origin domestication model for rice with subsequent introgression events: *geng* subspecies arose directly from wild progenitors in the Yangtze Basin, whereas *xian* emerged through hybridization between early domesticated *geng* and local wild populations [6,38,45]. This dual-path domestication underscores the dynamic interplay between human selection and natural genetic exchange in crop evolution.

Pigmented rice, primarily comprising red and black rice, accounts for a relatively small proportion of rice germplasm resources but possesses higher nutritional value. It is well-established that wild rice (*O. rufipogon*) consistently has a red pericarp due to proanthocyanidin accumulation. During rice domestication, loss-of-function mutations in the *Rc* gene—primarily a 14-bp deletion in exon 6 (the recessive *rc* allele)—resulted in the non-pigmented pericarp characteristic of white cultivated rice. This *rc* allele is fixed in most modern cultivated rice as a key domestication allele, having originated before the divergence of major rice subspecies [13].

Despite strong selection for white pericarp in cultivated rice, red pericarp persists in some varieties through several mechanisms: direct inheritance from wild ancestors; re-domestication of weedy rice, which is the process of harnessing beneficial alleles (such as those for stress resilience) from these de-domesticated populations; and continuous human selection driven by nutritional advantages or adaptive benefits in certain regions [46].

**Figure 7.**
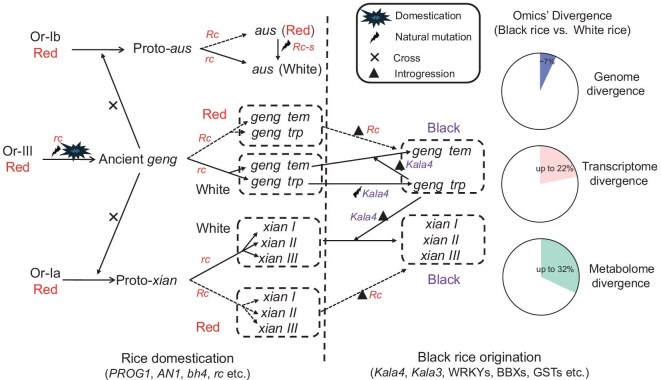
Evolutionary trajectory of black rice and its multi-omics divergence from white rice. Pericarp color is indicated by text labels (‘red’, ‘white’, ‘black’).

In contrast, black rice emerged later in rice evolution, arising from a gain-of-function mutation in the *OsKala4* gene, enabling anthocyanin biosynthesis. Our genomic evidence indicates that black rice originates from white rice: only 5.4% of black rice accessions carry a functional *OsRc* allele, while the rest carry the domesticated *rc* allele with the 14-bp deletion in exon 6. This finding suggests that black rice originated through post-domestication improvement. The rare functional *OsRc* alleles observed in black rice populations are likely introduced via introgression from red rice (Fig. 7).

Taken together, red rice represents an ancient wild-type trait inherited from ancestral rice, characterized by the accumulation of proanthocyanidins and the absence of anthocyanin production. By contrast, black rice originated through post-domestication improvement and, as a result, has acquired the ability to accumulate anthocyanins, irrespective of proanthocyanidin presence. This evolutionary and biochemical distinction offers a clear, trait-based classification criterion for differentiating between these two types of pigmented rice.

Genome-wide scans identified 238 differentially selected regions between black and white rice. We propose that these regions in black rice are functionally associated with pericarp pigmentation, as this trait represents the most pronounced phenotypic divergence between the two rice groups. This hypothesis can be supported by two lines of evidence: (i) early developmental uniformity: at 5 DAF, when anthocyanin biosynthesis remains inactive, transcriptomic and metabolomic divergence between black and white rice was minimal (0.61% DEGs and 0.54% DAMs), with divergence escalating rapidly from 10 DAF onward, coinciding with anthocyanin pathway activation; (ii) functional validation: transcription factors (e.g. *OsBBX13, OsWRKY1*) within these selected regions were experimentally confirmed to regulate anthocyanin biosynthesis, directly linking their selection to pigmentation. These selected regions drive extensive transcriptomic reprogramming (up to 22% DEGs during seed development) and metabolomic remodeling (up to 32% DAMs during seed development). However, many DEGs/DAMs showed no direct linkage to anthocyanin or flavonoid pathways. This could be explained by either genetic hitchhiking of unrelated alleles during differential selection between black and white rice varieties, or pleiotropic effects of pigmentation-related regulators. For example, *OsMYB3*, a black rice-specific pigmentation gene, has been reported to regulate both anthocyanin biosynthesis and grain size [30,47], demonstrating unintended trait correlations. These findings underscore a critical challenge in black rice breeding: balancing nutritional enhancement (achieved through anthocyanin enrichment) with essential agronomic traits. This highlights the necessity for precision breeding strategies that can decouple nutritional improvement from essential agronomic characteristics during cultivar development.

The MBW transcriptional complex (MYB-bHLH-WD40) serves as a central regulatory module for flavonoid biosynthesis across plant species, directly activating anthocyanin biosynthetic pathways [48,49]. In rice, canonical MBW components—including MYB (OsC1, OsPL, OsMYB3), bHLH partners (OsKala4, OsRb, OsPa, OsPs) and WD40 scaffold (OsTTG1)—have been systematically characterized through map-based cloning and multi-omics approaches [14,30,31,33,48–51]. Our study extends this paradigm by identifying three previously uncharacterized regulators (OsWRKY1, OsBBX13 and OsTT12) that orchestrate anthocyanin synthesis and compartmentalization in black rice through integrated multi-omics profiling and GWAS (Table S10). Mechanistically, OsBBX13 physically interacts with the MBW component OsMYB3, while OsWRKY1 interacts with OsTTG1, synergistically amplifying the transcriptional activation of key anthocyanin biosynthetic genes (e.g. *OsF3′H, OsDFR, OsANS*) (Fig. 3d–h).

Synthesized on the cytoplasmic surface of the endoplasmic reticulum (ER), anthocyanins are transported to the vacuole for storage. This process relies on a coordinated multi-protein system: cytosolic GSTs escort pigments to the tonoplast, where MATE and ABC transporters then mediate their final import into the vacuolar lumen [52]. Several rice transporters have been validated to participate in anthocyanin transport. For instance, simultaneous knockout of *OsGSTU34* (a Tau-class GST), *OsMRP15* (a C-type ABC transporter) and *OsAnP* (an *AtTT12* homolog) results in loss of purple pigmentation in leaves [53]. Mackon *et al.* further confirmed that knockout of *OsGSTU34* alone in black rice leads to a significant reduction in anthocyanin accumulation across various tissues, including seeds [54]. In the present study, knockout of the vacuolar transporter OsTT12 causes only a partial reduction in seed anthocyanins, suggesting functional redundancy with other transporters, likely the tonoplast-localized OsMRP15, unlike the cytoplasmic OsGSTU34. The possibility that other rice transporters are also involved in anthocyanin transport cannot be ruled out. The rice genome is estimated to harbor approximately 62 GSTs [55], 46 MATEs [56] and 12 multidrug resistance-associated proteins (MRPs) [57]. Notably, several members, including *OsGSTU6, OsGSTU38, OsGSTU45, OsMATE31* and *OsMATE55* are located within genomic regions under differential selection between black and white rice. Nevertheless, the full complement of transporters participating in anthocyanin sequestration and their functional relationships requires further systematic investigation.

Moreover, transcriptional activation assays in protoplasts demonstrated that the MBW complex upregulates the expression of *OsWRKY1* and *OsBBX13* (Fig. S14a). Consistent with a role in anthocyanin biosynthesis under MBW regulation, the expression patterns of *OsWRKY1* and *OsBBX13* closely resembled those of *OsKala4* and the MBW-activated anthocyanin biosynthetic genes, showing significantly higher expression in black rice seeds compared with white rice seeds (Fig. 3a and b). In contrast, *OsTT12* appears to play a universal role in rice, as its expression was not significantly regulated by the MBW complex, OsWRKY1, OsBBX13 or their associated complexes in our assays (Fig. S14b). This is consistent with the distinct expression profile of *OsTT12*, which differs from that of anthocyanin biosynthetic genes and shows no significant differential expression between black and white rice seeds (Fig. S14c). Collectively, our findings reveal a multi-layered regulatory mechanism that fine-tunes anthocyanin flux in black rice, with OsWRKY1 and OsBBX13 integrated into the MBW hierarchy, while OsTT12 functions in a parallel, constitutive manner.

Relatively low yield is a major constraint for the widespread cultivation of black rice. Although the underlying mechanisms are not fully elucidated, several hypotheses have been proposed: (i) competition for metabolic resources due to the substantial energy and carbon sink strength of anthocyanin biosynthesis; (ii) reduced photosynthetic efficiency caused by anthocyanin accumulation in the pericarp and other vegetative tissues (e.g. leaf, hull), which can shade chloroplasts and potentially lower chlorophyll content; and (iii) inherent agronomic weaknesses in many black rice varieties, such as susceptibility to lodging, late maturity and high vulnerability to blast disease (*Magnaporthe oryzae*), leading to additional yield losses [36]. The first two reasons are directly linked to anthocyanin biosynthesis. Our previous research demonstrated that the MYB transcription factor gene *OsC1* specifically determines anthocyanin synthesis in leaves; its loss of function eliminates leaf pigmentation without affecting anthocyanin synthesis in seeds [31]. Therefore, minimizing anthocyanin accumulation in vegetative tissues, especially leaves—the primary organ for photosynthesis—is a viable strategy to alleviate the yield penalty in black rice without compromising its nutritional value. The third reason, however, appears to be related to the low level of breeding efforts for black rice. This is because white rice dominates the market, leading most breeders to focus solely on the development of white rice varieties.

In this study, evaluation of agronomic traits using two pairs of black and white rice sister lines confirmed that the black rice lines had a yield disadvantage compared to their white counterparts. However, the two pairs of sister lines differed in their genetic similarity (98.36% vs. 96.02%), and the pair with a higher similarity exhibited a much smaller yield gap (Fig. S13b–S13d). This suggests that the yield reduction in black rice is at least partly attributable to the linkage between pericarp color genes and unintended, yield-penalizing alleles in the genetic background, since minimizing genetic differences appears to mitigate the yield gap between black and white rice.

Furthermore, grain width, grain weight and seed setting rate were consistently and significantly lower in both black rice lines compared to their white counterparts. These three traits likely represent a physiological/metabolic cost associated with anthocyanin biosynthesis in black rice, potentially caused by anthocyanin biosynthesis itself or linkage drag. Consequently, targeted improvement of these traits presents a promising strategy for reducing the yield gap. Our analysis of the selection status for the 225 QTNs within the black rice collection will directly facilitate this targeted breeding approach.

In conclusion, this study constructs a global black rice germplasm resource comprising 367 accessions, and employs integrated multi-omics approaches (genomics, transcriptomics, metabolomics) to systematically dissect the genetic and molecular divergence between black and white rice. Population genomic analyses unveil a two-step domestication trajectory: initial loss of proanthocyanidin pigmentation through *Rc* inactivation, followed by *OsKala4*- and *OsMYB3*-driven anthocyanin pathway activation under artificial selection. Multi-omics profiling identifies 238 differentially selected loci that drive substantial transcriptomic and metabolomic remodeling, with functional validation demonstrating that newly identified fine tuners (*OsWRKY1, OsBBX13, OsTT12*) synergistically enhance the MBW transcriptional complex to coordinate regulation of anthocyanin biosynthesis and vacuolar sequestration. Furthermore, the analysis of QTN selection status and agronomic trait differences between black and white rice provides valuable insights for the targeted genetic improvement of black rice varieties. These findings not only advance our understanding of pigmentation evolution in black rice but also provide a molecular roadmap for precision breeding programs to decouple nutritional enhancement from agronomic trade-offs, thereby facilitating the development of elite black rice cultivars with optimized nutritional and agronomic performance.

## MATERIALS AND METHODS

### Genome sequencing of black rice collection

A global collection of 367 black rice accessions was sequenced (∼20× coverage). Clean reads were aligned to the *Nipponbare* reference genome (IRGSP-1.0). Approximately 4.3 million high-confidence SNPs were identified using the Genome Analysis Toolkit (GATK) [58].

### Population genetics analysis

A neighbor-joining phylogenetic tree was reconstructed based on a pairwise genetic distance matrix computed with PLINK [59] and visualized in MEGA [60] and iTOL [61]. Population structure was inferred using ADMIXTURE [62], and PCA was performed with GCTA [63]. Selective sweeps were identified using XP-CLR [64]. *F*_st_ and *π* were calculated in 10-kb windows using VCFtools [65]. Haplotype networks were constructed from high-quality SNPs after variant imputation and phasing with Beagle [66], and visualized using Haplotype Viewer. RiceNavi software was employed to identify QTNs [37].

### Transcriptomic and metabolomic analysis

Transcriptomic and non-targeted metabolomic analyses were conducted using developing seeds of black and white rice collected at 5, 10, 15 and 20 DAF. RNA-Seq was conducted by the Smartgenomics Technology Institute (Tianjin, China), and the transcriptomic data were processed to identify DEGs using DESeq2 [67]. Non-targeted metabolomic analysis was conducted by the BioTree Biotech Co. (Shanghai, China), and DAMs were identified through OPLS-DA. Additionally, targeted anthocyanin quantification was performed on mature seeds via LC-MS/MS, with the multiple reaction monitoring (MRM) transitions for each analyte provided in Table S11. Finally, an integrated co-expression network was constructed by calculating Pearson’s correlations (∣*r*∣ > 0.3) among metabolites, biosynthetic genes and transcription factors, and visualized using Gephi [68].

### *In vitro* gene function analysis

Protein–protein interactions were tested using a yeast-two-hybrid assay in AH109 cells and an LCI assay [69]. For subcellular localization, the coding sequences of candidate genes were fused to mCherry in the pM999 vector, transiently expressed in rice protoplasts, and imaged by confocal microscopy. The dual-luciferase assay was conducted in rice protoplasts, measuring the activation of anthocyanin gene promoters (e.g. *OsCHS, OsDFR*) by candidate regulators. The *in vitro* transport of anthocyanin substrates by OsTT12 was assayed in a yeast system according to Zhao *et al.* [70].

### GWAS

A GWAS was performed using factored spectrally transformed linear mixed models provided by the FaST-LMM program [71].

### Knockout and overexpression transformation

The CRISPR/Cas9 knockout vectors were constructed according to Ma *et al.* [53]. The full-length cDNA sequences of *OsWRKY1, OsBBX13* and *OsTT12* were isolated from black rice for overexpression vector construction. *Agrobacterium*-mediated genetic transformation of black rice was performed following the protocol of Lin *et al.* [72]. All primers are listed in Table S12.

### Development and agronomic comparison of black and white rice sister lines

Two pairs of black and white rice sister lines from separate crosses were developed, and their key agronomic traits were compared in a field trial following a randomized block design.

For more details on the Materials and Methods, please refer to the Supplementary data.

## Supplementary Material

nwaf497_Supplemental_Files

## Data Availability

The raw datasets used for whole-genome resequencing, RNA-Seq, were deposited into the China National Center for Bioinformation (CNCB) SRA database under the accession numbers PRJCA044699 and PRJCA026531.
