# Supplementary material for: Integrative multi-omics analysis reveals the domestication mechanism of black rice
Source: Natl Sci Rev. 2025 Nov 13;13(10):nwaf497. doi: 10.1093/nsr/nwaf497 (PMC13247721; doi:10.1093/nsr/nwaf497)
Supplement: nwaf497_Supplemental_Files [file nwaf497_supplemental_files.zip › Supplementary Figures.docx]

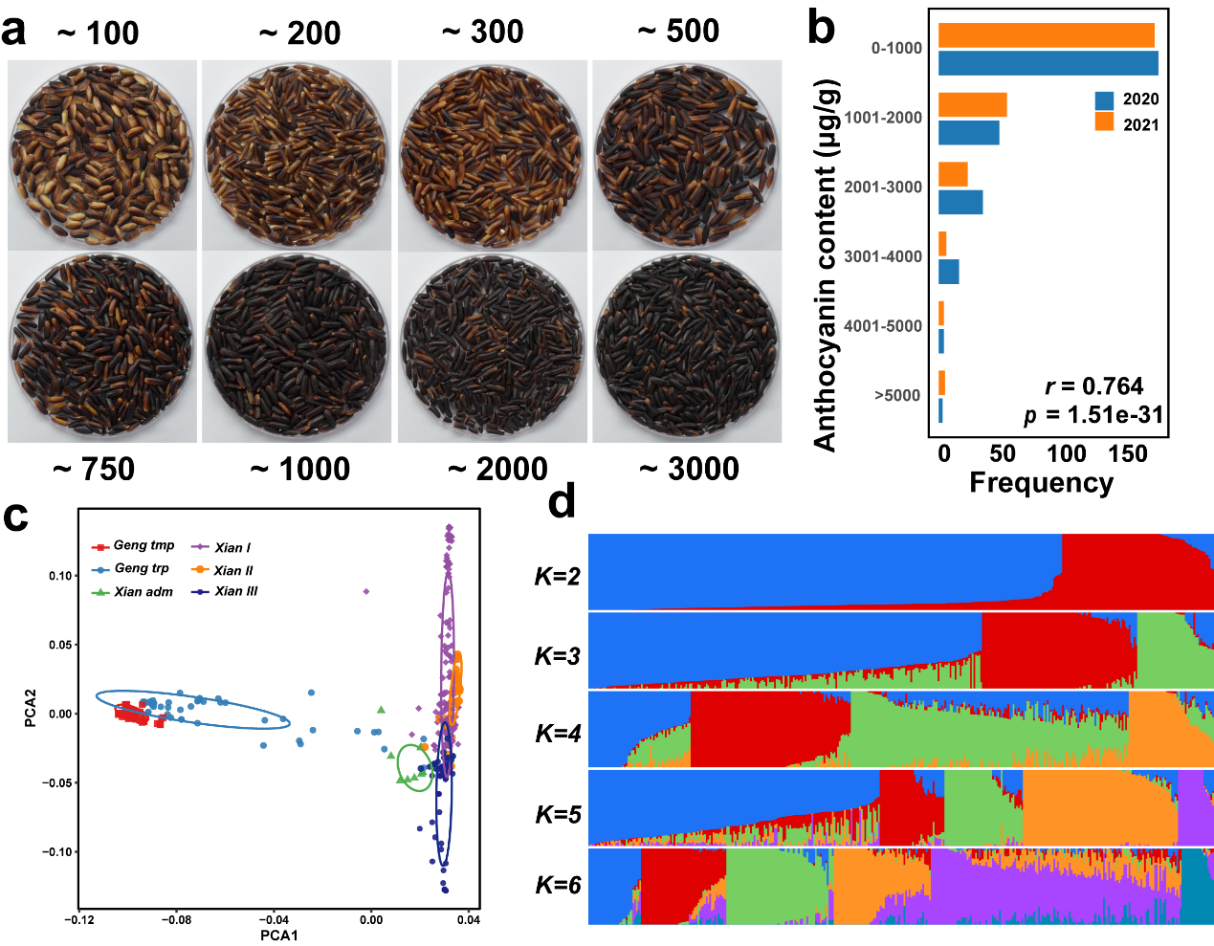


**Fig. S1** Population genetic analysis of black rice population. (a) Appearance of black rice grains with various anthocyanin contents (μg/g DW). (b) Distribution of anthocyanin content in the black rice population in 2020 and 2021. The Pearson’s correlation coefficient (*r*) and its statistical significance between the two years are indicated. (c) Principal component analysis of black rice. (d) Population structure inferred by ADMIXTURE analysis (K = 2–6).


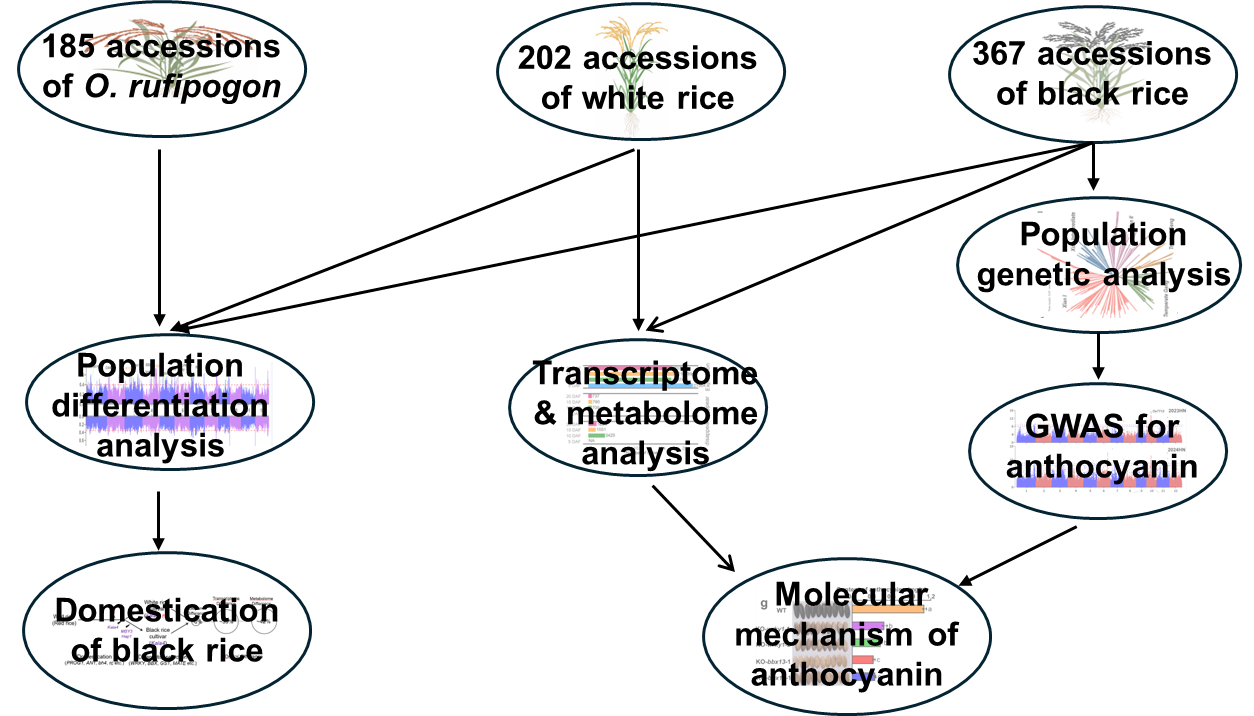


**Fig. S2** Schematic of the experimental design employed in this study.


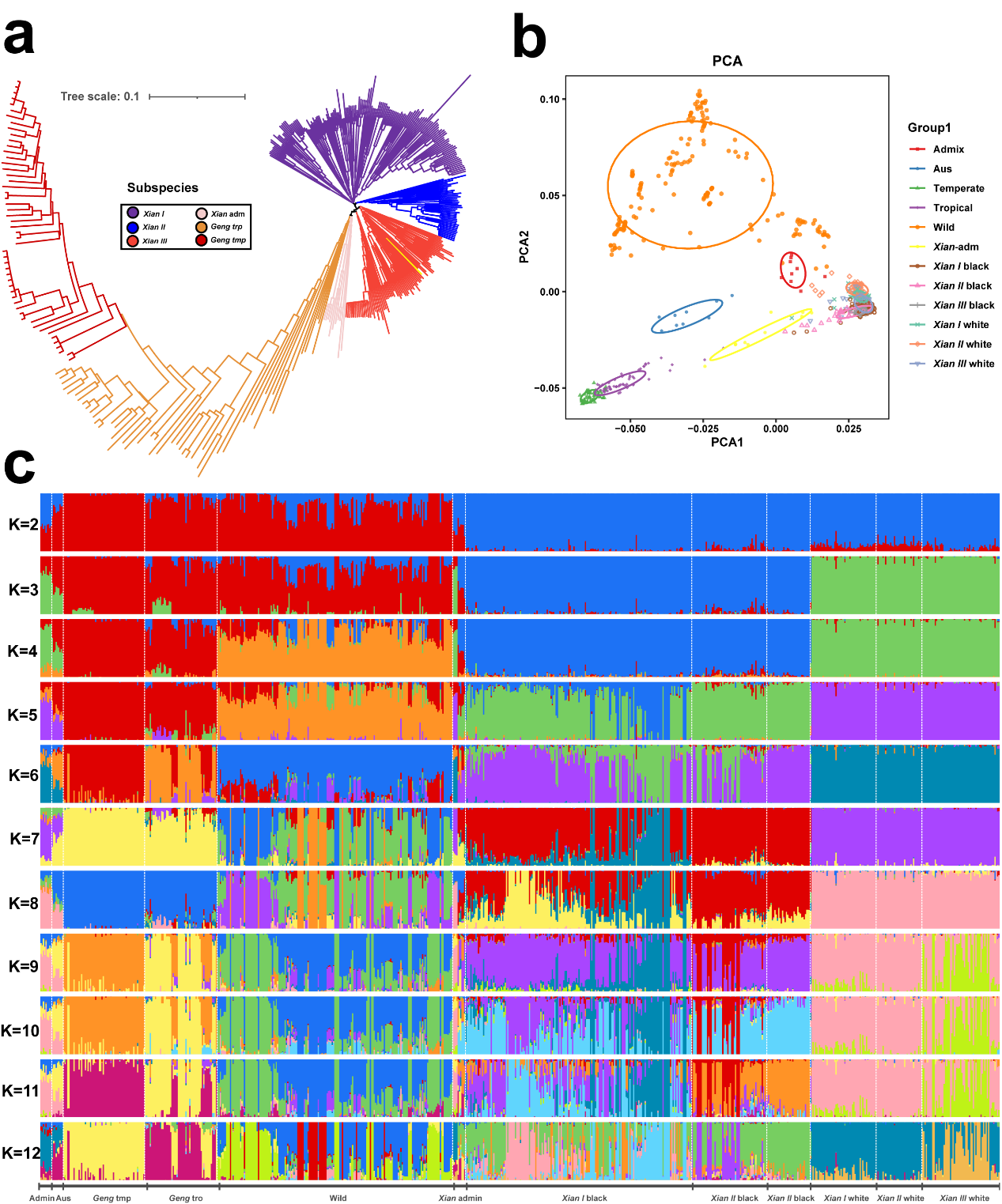


**Fig. S3** Population genetic analysis of all 754 rice accessions. (a) Neighbor-joining tree of 367 black rice accessions, identifying six groups: *Xian I*, *Xian II*, *Xian III*, *Xian adm*, *temperate* *Geng* and *tropical* *Geng*. (b) Principal component analysis of all 754 rice accessions. (c) ADMIXTURE plot for non-admixed rice accessions (subset of 754 accessions).


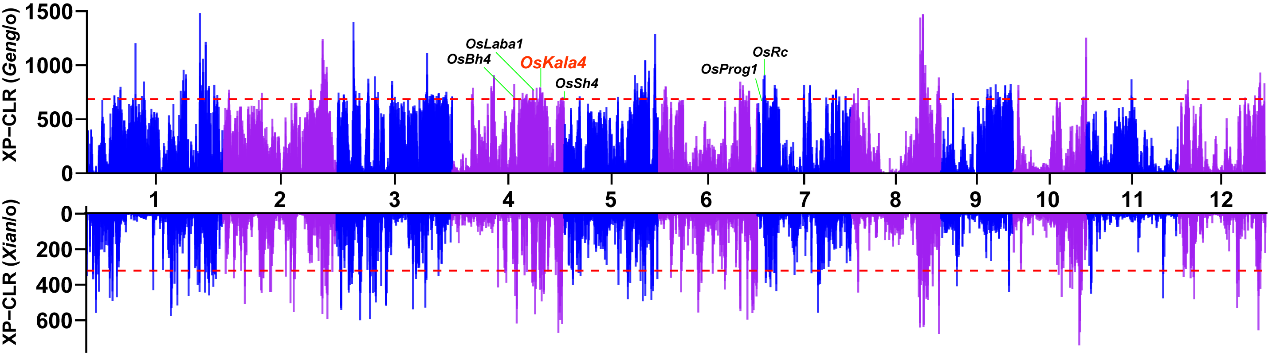


**Fig. S4** Genome-wide scan of selection signals in *Geng* black and *Xian* black population using the XP-CLR method. Horizontal dashed lines indicate genome-wide significance thresholds (XP-CLR score > 685.93 and > 320.75). Well-characterized domestication loci are highlighted in black.


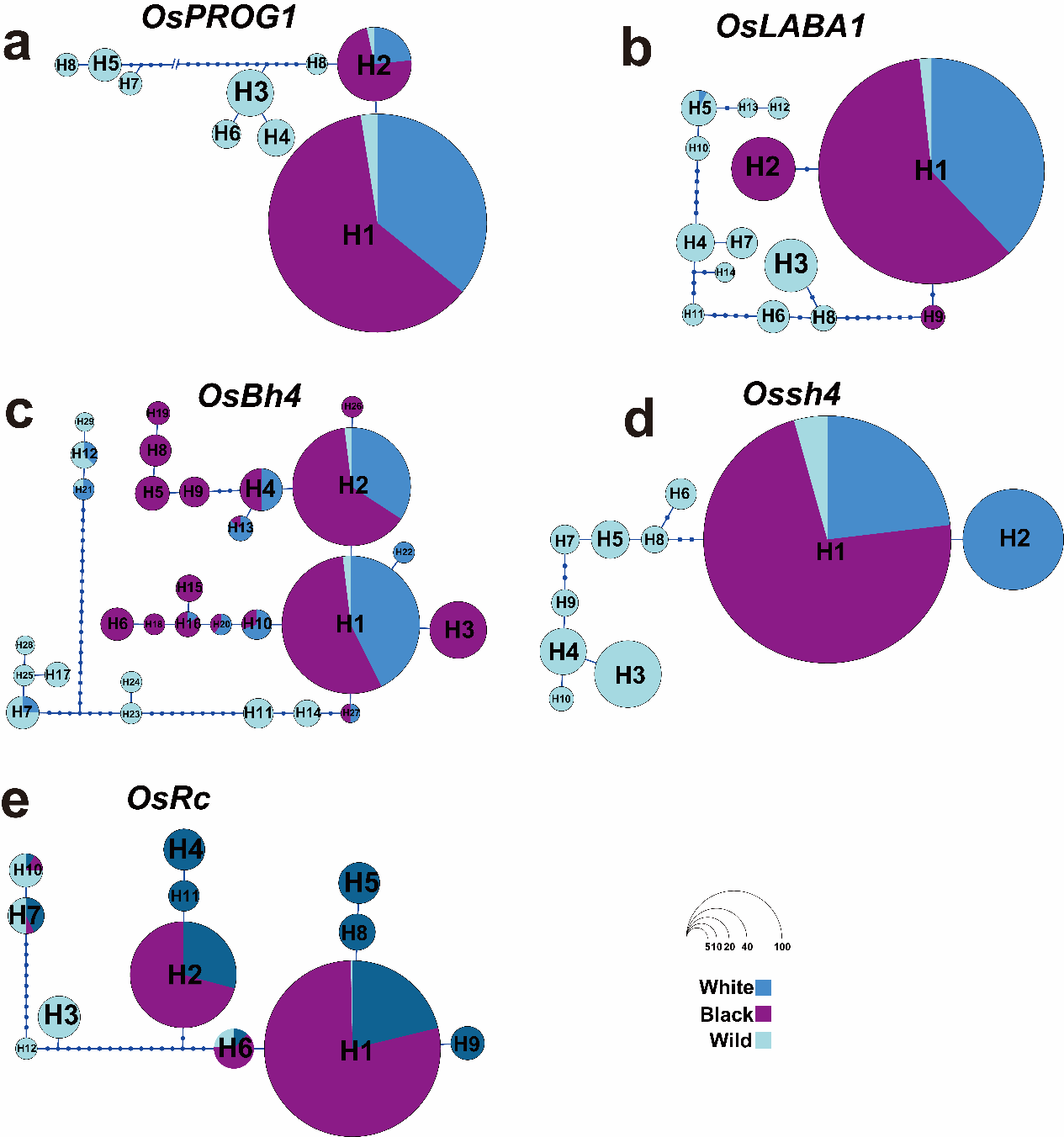


**Fig. S5** Haplotype networks of representative domestication genes. (a) *OsPROG1*, (b) *OsLABA1*, (c) *OsBh4*, (d) *Ossh4*, (e) *OsRc*. Circle size corresponds to haplotype frequency.

**
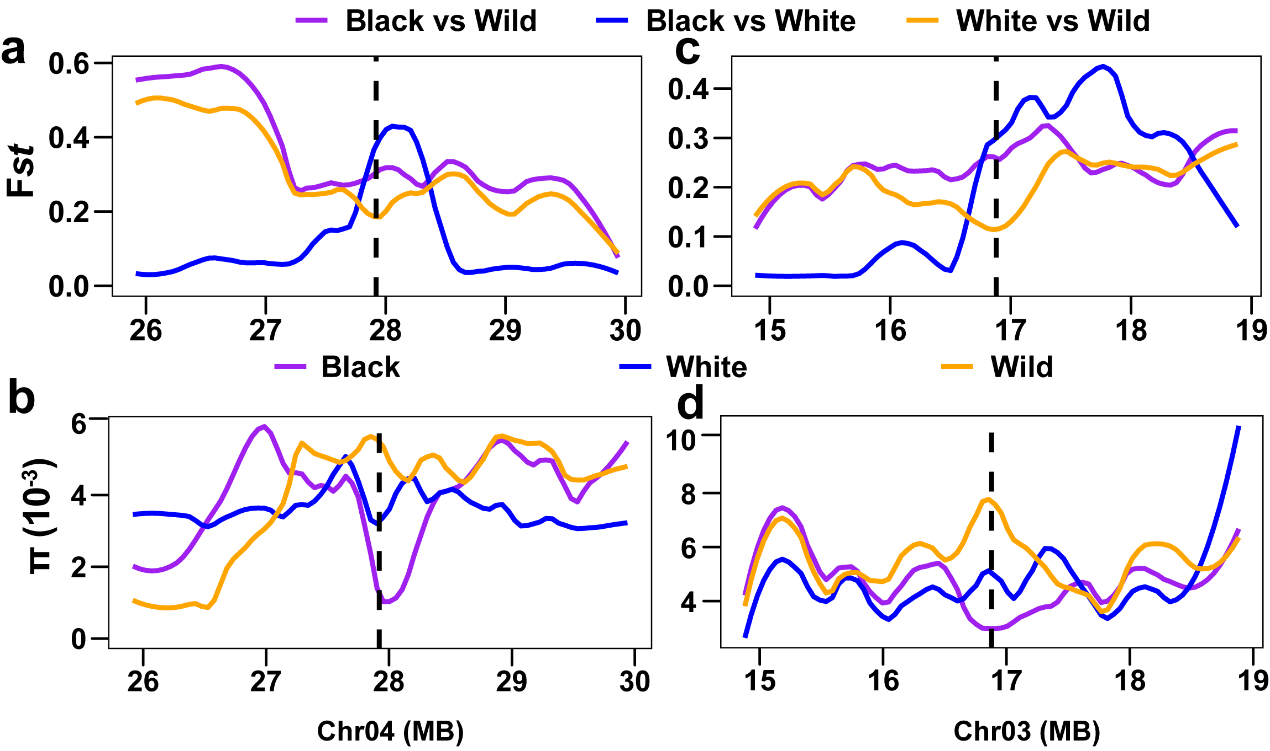
**

**Fig. S6** Population genetics differentiation (*Fst*) and Nucleotide diversity (π) around the *OsKala4* and *OsMYB3* gene. (a) F*st* distribution between black vs. wild and white vs. wild population within a 4-Mb region flanking *OsKala4*. (b) Nucleotide diversity (π) among black rice, white rice, and wild rice population in the same 4-Mb region. The black dashed line indicates the position of *OsKala4.* (c) F*st* distribution between black vs. wild and white vs. wild population within a 4-Mb region flanking *OsMYB3*. (d) Nucleotide diversity (π) among black rice, white rice, and wild rice population in the same 4-Mb region. The black dashed line indicates the position of *OsMYB3.*


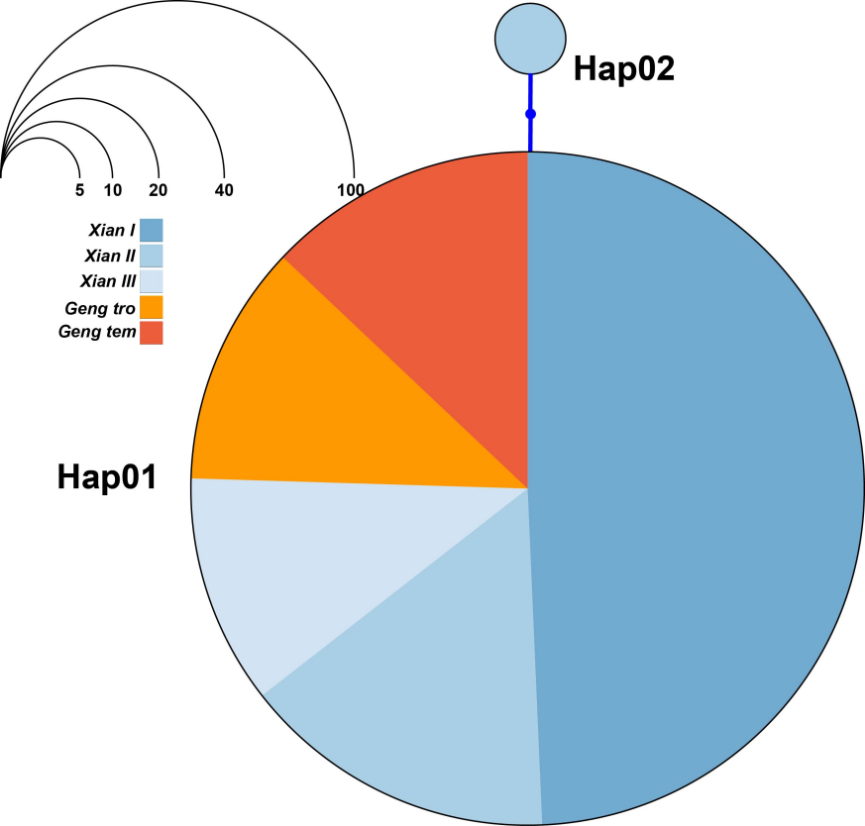
**Fig. S7** Haplotype analysis of the *Kala4* promoter region across the black rice population.


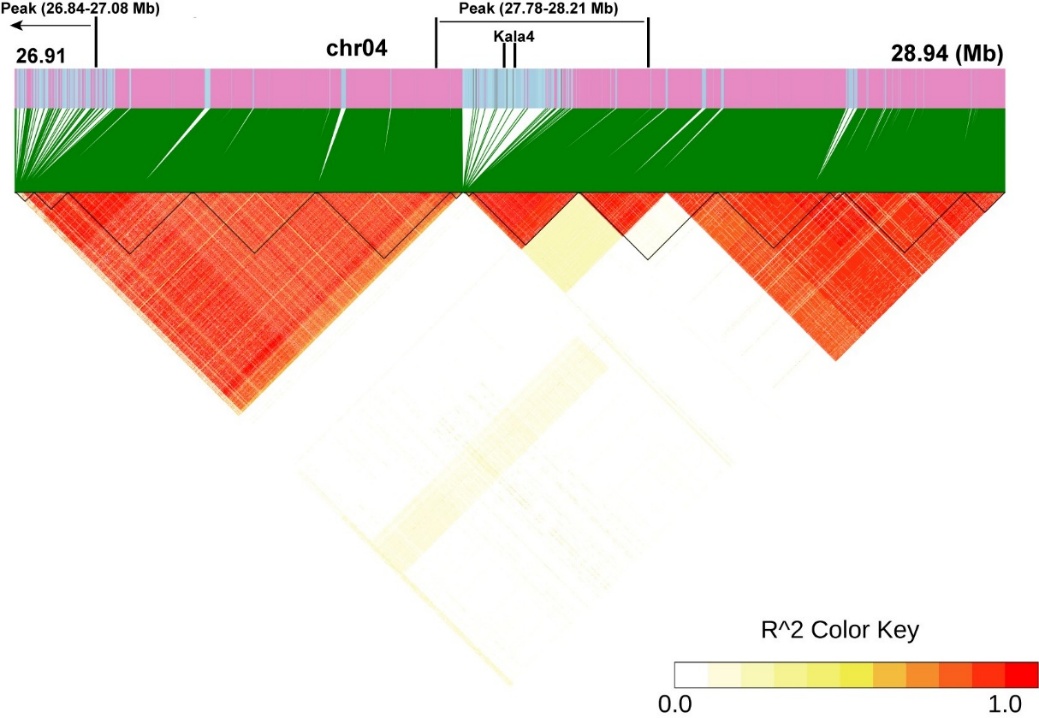


**Fig. S8** Assessment of linkage between *Kala4* and its flanking genomic regions.


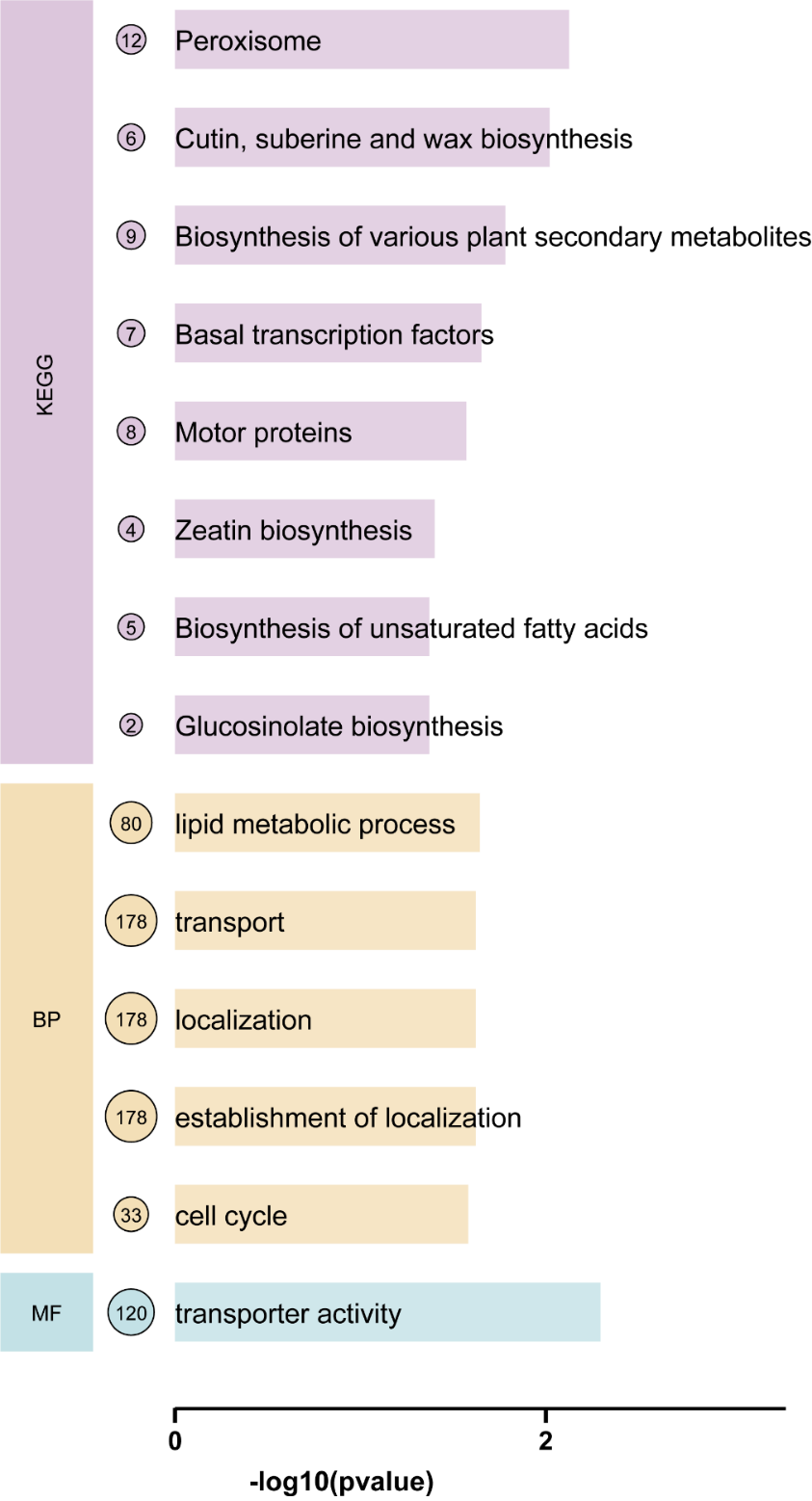


**Fig. S9** Functional enrichment analyses of KEGG pathways and Gene Ontology (GO) terms for 2,656 differentially selected genes between black and white rice identified by XP-CLR scanning. Enrichment scores represent the significance of gene associations with specific biological processes.

**
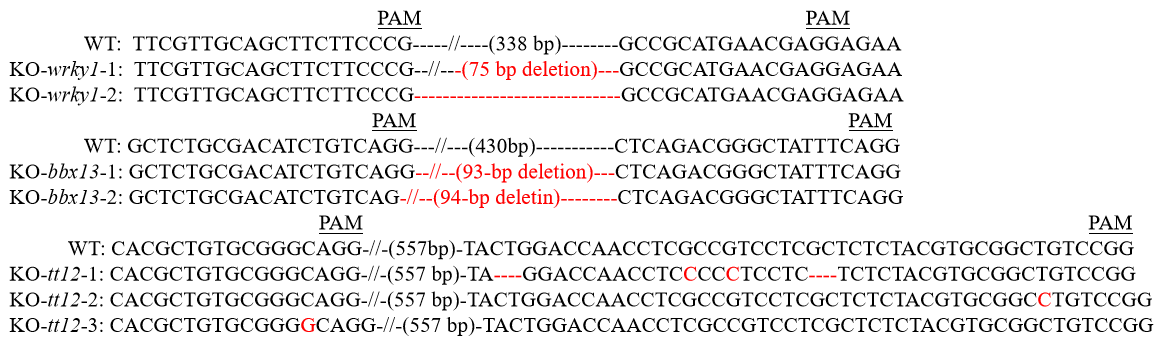
**

**Fig. S10** Sequencing confirmation of mutations in *OsWRKY1*, *OsBBX13*, and *OsTTG1* knockout lines generated by CRISPR/Cas9 in the W110 background.


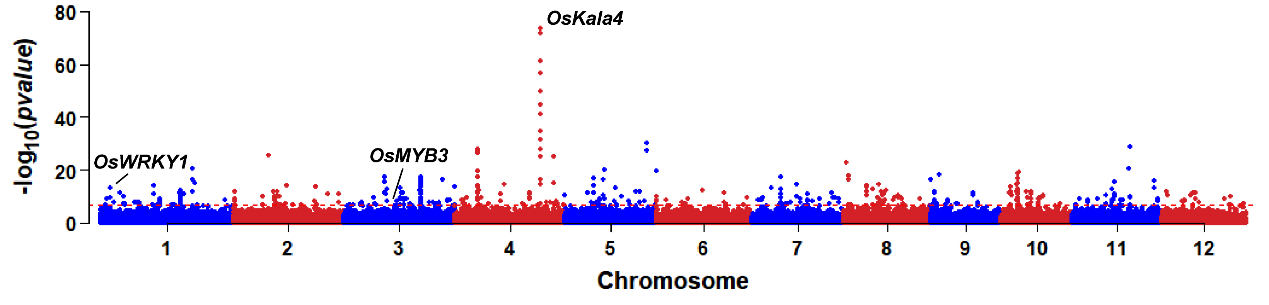


**Fig. S11** Genome-wide association study for black pericarp in a mixed rice population (367 black rice accessions and 202 white rice accessions).


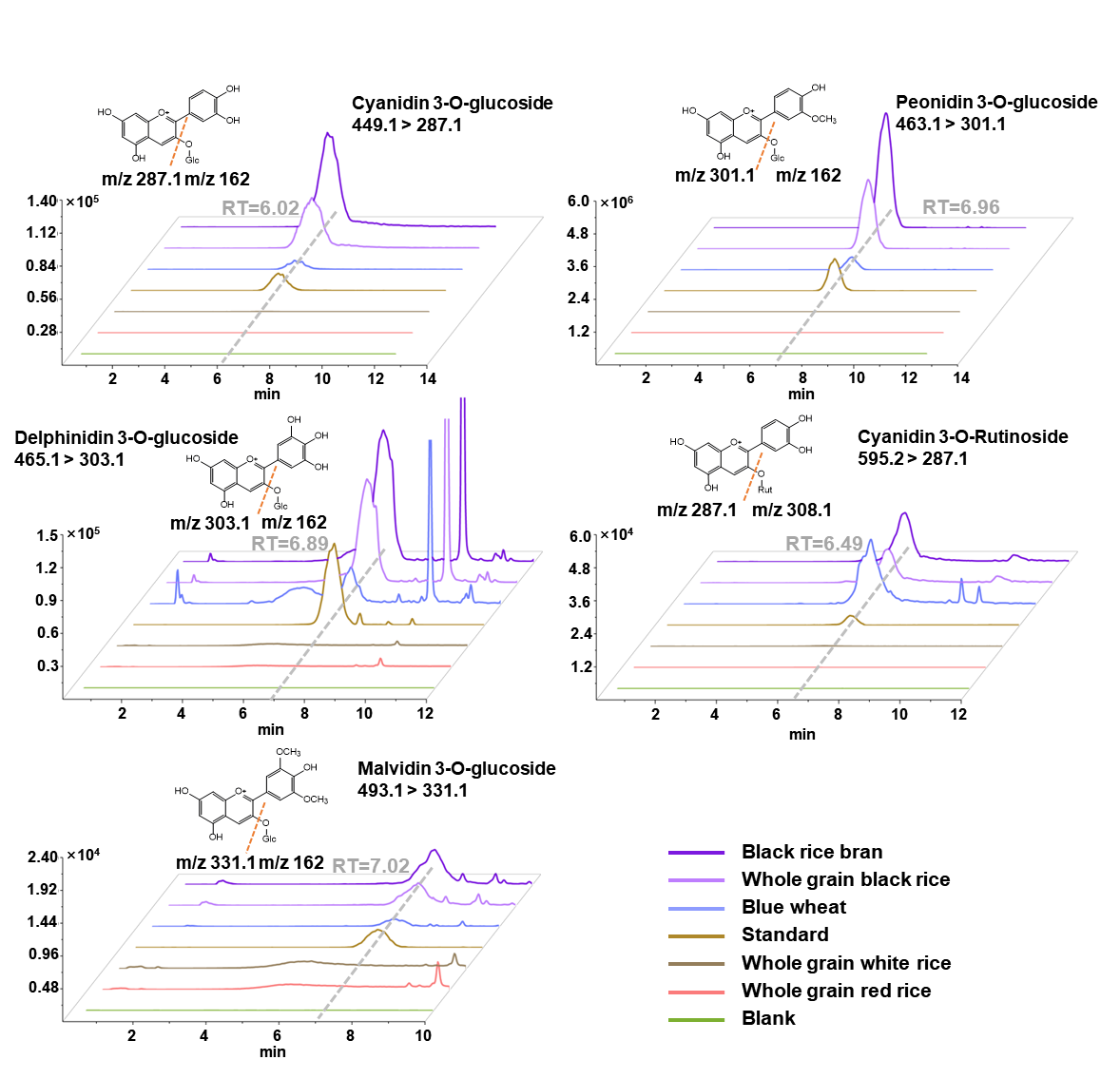


**Fig. S12** Targeted metabolite analysis of five anthocyanins cyanidin 3-O-glucoside, cyanidin 3-O-rutinoside, peonidin 3-O-glucoside, delphinidin 3-O-glucoside, and malvidin 3-O-glucoside in black rice samples with authentic standards.


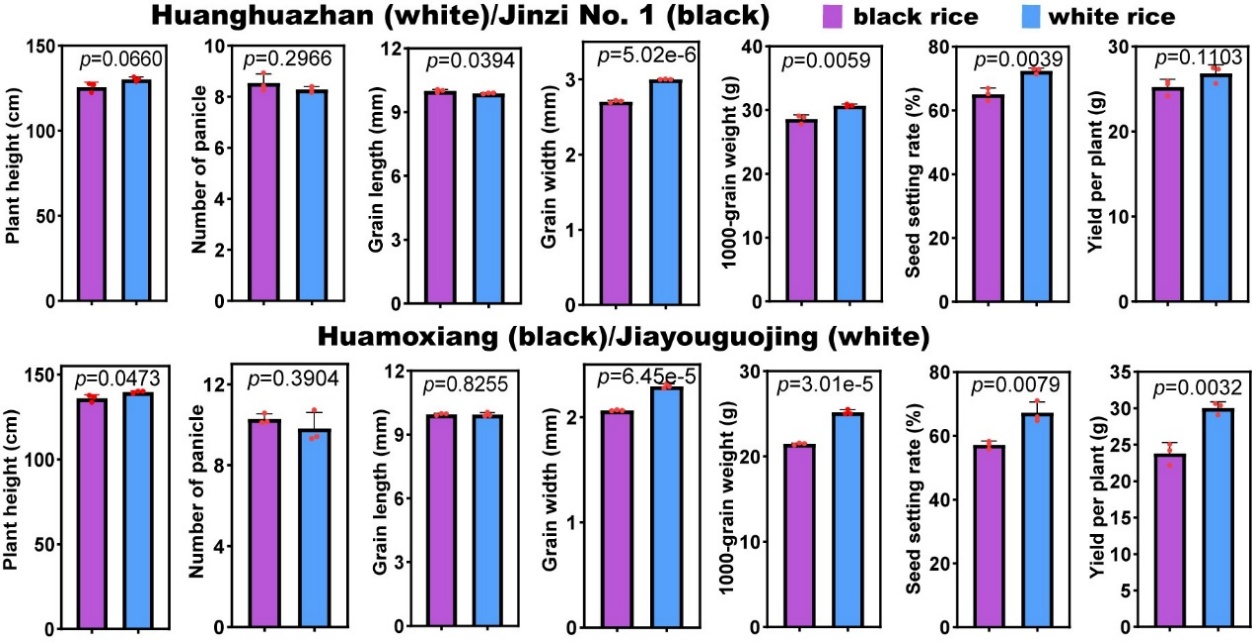


**a**


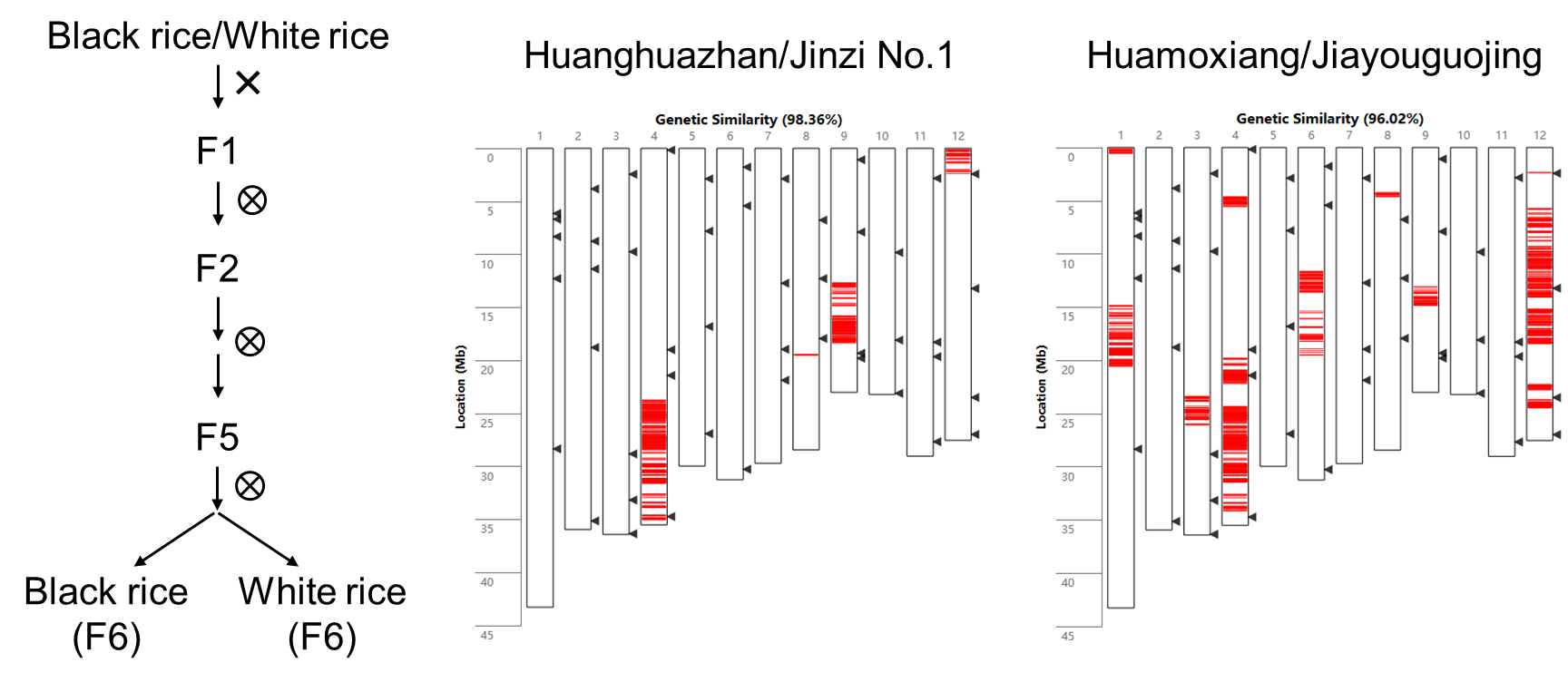


**b**

**c**

**d**

**Fig. S13** Development and agronomic trait evaluation of black rice and white rice sister lines. (a) Developmental process of black rice and white rice sister lines. (b) Genetic similarity of black rice and white rice sister lines derived from the cross between Huanghuazhan (white rice) and Jinzi No.1 (black rice). (c) Genetic similarity of black rice and white rice sister lines derived from the cross between Huamoxiang (black rice) and Jiayouguojin (white rice). (d) Comparison agronomic traits between two pairs of black rice line and white rice line. Each point on the bars represents the mean value of a plot containing 40 plants (n = 3). All data indicate mean ± SD. Statistical analysis was performed according to the two-tailed Student’s *t*-test.

**
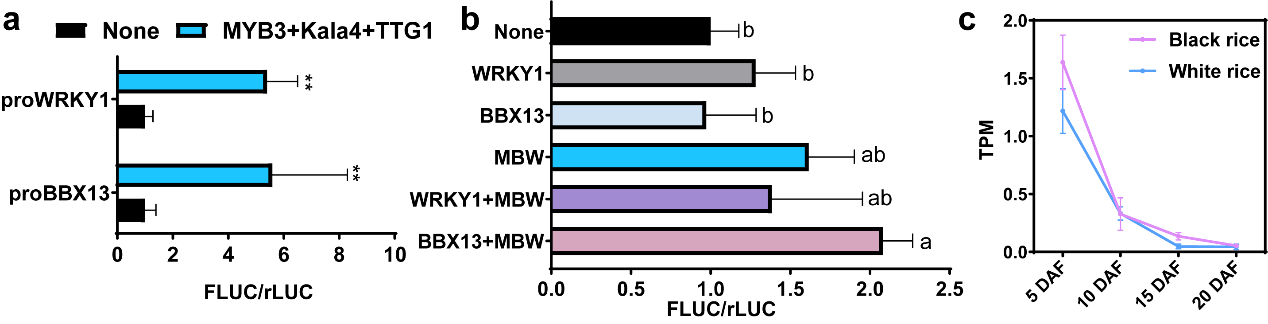
**

**Fig. S14** Transcriptional regulation analysis of *OsWRKY1*, *OsBBX13* and *OsTT12*. (a) Transcriptional activation of *OsWRKY1* and *OsBBX13* by the MBW complex, as determined by dual-luciferase assays. ** represent significant difference at *p* < 0.01 according to the two-tailed Student’s *t*-test (n = 5). (b) Transcriptional activation of *OsTT12* by OsWRKY1, OsBBX13, the MBW complex, and their combinations. Different lowercase letters represent significant difference at *p* < 0.05 according to the Tukey’s honest significant difference test (n = 5). (c) Expression levels of *OsTT12* during seeds development. All data indicate mean ± SD.
