# Supplementary material for: Integrative multi-omics analysis reveals the domestication mechanism of black rice
Source: Natl Sci Rev. 2025 Nov 13;13(10):nwaf497. doi: 10.1093/nsr/nwaf497 (PMC13247721; doi:10.1093/nsr/nwaf497)
Supplement: nwaf497_Supplemental_Files [file nwaf497_supplemental_files.zip › Supplementary Materials and Methods.docx]

**Materials and Methods**

**Black rice collection and genomic sequencing**

A total of 367 black rice accessions were collected worldwide and sequenced at an average depth of ~ 20×. Young seedling of each black rice accession was collected and their genomic DNA was extracted with the cetyl trimethylammonium bromide (CTAB) method [1]. Genome sequencing libraries were constructed and sequenced on an Illumina HiSeq 2000 platform, generating ~ 20.3 billion 150-bp paired-end reads for all samples.

**Mapping and variation calling**

Raw sequencing data were initially processed using fastp (version 0.23.0) [2] to remove adapter sequences and low-quality reads. Clean reads from all rice varieties were then independently aligned to the *Nipponbare* reference genome (IRGSP-1.0) using BWA (version 0.7.17) with parameters “mem -t 4 -k 32 -M -R” [3]. Sequencing coverage and accession-specific metrics are provided in Table S1.

SNP calling was performed with GATK (version 4.1.2) HaplotypeCaller tool, which conducted local *de novo* haplotype assembly within active genomic regions. Variants were filtered using the following criteria “QUAL < 30.0 || AC < 10 || DP < 50 || QD < 2.0 || MQ < 30.0 || FS > 60.0 || SOR > 3.0 || MQRankSum < -12.5 || ReadPosRankSum < -8.0”[4]. Subsequent filtering excluded SNPs with minor allele frequency (MAF ≤ 0.05) and genotype call rate < 90% (missing data > 10%). This pipeline ultimately identified approximately 4.3 million high-confidence SNPs. RiceNavi software was employed to identify QTNs associated with various phenotypic traits [5].

**Phylogenetic analysis and population structure**

To obtain reliable results for the population history of mulberry, SNPs were first filtered by removing those with low MAF (≤0.05) and low genotype rate (≤0.1). To reconstruct neighbor-joining phylogenetic trees using SNP data, we estimated the pairwise genetic distance matrix using PLINK (version 1.90)[6] with the parameters “-distance 1-ibs flat-missing” and reconstructed the trees on the basis of the distance matrix using MEGA (version 10.1.0)[7]. The midpoint rooting was calculated and tree branches of the final tree were coloured in iTOL (https://itol.embl.de/) [8].

The population genetic structure of wild and domesticated rice was explored using ADMIXTURE (version 1.3.0)[9] on the basis of the LD-pruned pseudomolecule SNP data with the parameter “-indep-pairwise 200 100 0.1” in PLINK.

PCA was performed to study relatedness and clustering among populations or samples using GCTA software (version 1.94.4) [10] with the parameter “-pca 20” to output the first 20 and all eigenvalues.

**Selective sweep analysis**

We used XP-CLR for selective sweep analysis. XP-CLR was performed using the python version of XP-CLR (version 1.1.2) [11] with the parameters “-maxsnps 200 --size 100 kb --step 10 kb”. We similarly used the top 1% window as the candidate region for selective scanning analysis. Then, the genes within final candidate regions were defined as selective sweep candidate genes, and enrichment analysis was performed on these selective sweep candidate genes using the GO (Gene Ontology) and KEGG databases.

Population differentiation statistic (F*st*) and nucleotide diversity (π) were used to detect selective signatures between different groups in 10-kb windows using VCFtools (version 0.1.16) software [12]. Specifically, the π value for each gene was calculated using vcftools software to analyze genetic variation data in the region spanning 2 kb upstream to 1 kb downstream of the gene.

**Haplotype network analysis**

We first defined common haplotypes as those with frequencies > 0.5% and retained them for network reconstruction. To ensure variant quality, we filtered SNPs by removing those with minor allele frequency (MAF) <5% or missing rate >10%. We further refined the sample set by excluding accessions with >20% missing sites within the genomic region spanning 1 kb upstream to 1 kb downstream of the target gene. Subsequently, we used Beagle (version 5.4) [13] to impute and phase variants in the 400-kb region (200 kb upstream to 200 kb downstream of the gene) for this filtered sample set. Finally, we constructed median-joining networks using Haplotype Viewer (http://www.cibiv.at/~greg/haploviewer.shtml).

**Transcriptomic and non-targeted metabolomic analysis for developing rice seeds**

Representative black rice and white rice accessions were grown in the experimental farm of the Huazhong Agricultural University. Panicles were tagged at flowering date and harvested at 5 d, 10 d, 15 d, and 20 d after flowering. After isolated from harvested panicles, the seeds were immediately frozen in liquid nitrogen and then stored at -80 °C until measurement.

For transcriptomic analysis, the developing seed samples were first purified by using Fruit-mate™ for RNA Purification (Takara, Kyoto, Japan) to remove polysaccharide and then total RNA was extracted by using TransZol (TransGen, Beijing, China). RNA sequencing was conducted by the Smartgenomics Technology Institute (Tianjin, China). The transcriptome libraries of were sequenced using 150-bp paired-end Illumina sequencing with libraries of 300-bp insert sizes. After filtering out low-quality reads, the clean reads were uniquely mapped to the rice reference genome using HISAT2 (version 2.2.1) [14]. These high-quality alignments were then used to calculate genome-wide gene expression patterns. StringTie (version 2.1.4) was used to calculate the TPM for each gene among samples [15]. For cluster analysis of RNA-seq, we used the normalized matrix of TPM counts, the matrix was clustered using the visCluster function from clusterGvis (V.0.1.0) with six clusters (ClusterGVis: One-step to Cluster and Visualize Gene Expression Matrix. https://github.com/junjunlab/ClusterGVis). Differentially expressed genes (DEGs) were identified using DESeq2 (version 1.2.4R) [16] (|log2(FC)| >1 and Padj < 0.05). GO (including biological process, cellular component and molecular function) and KEGG pathway enrichment analyses of DEGs across the samples were performed through the clusterProfiler R package [17].

For metabolomic analysis, developing seed samples were subjected to non-targeted profiling at the Biotree Biotechnology Company (Shanghai, China). Seed samples (20 ± 1 mg) at different stages were taken and lyophilized, and mixed with beads and 1000 μL of extraction solution (MeOH:ACN:H_2_O, 2:2:1, v/v). The mixtures were then homogenized (35 Hz, 4 min) and sonicated for 5 min in 4 ℃ water bath; this step was repeated three times. The supernatant was transferred to a fresh glass vial for analysis. A quality control (QC) sample was prepared by combining equal aliquots of supernatant from each sample. Polar metabolites were analyzed using LC-MS/MS with an UHPLC system (Waters ACQUITY UPLC BEH Amide column, 2.1 mm × 50 mm, 1.7 μm) coupled to an Orbitrap Exploris 120 mass spectrometer (Thermo Scientific). The mobile phase consisted of (A) 25 mmol/L ammonium acetate and 25 mmol/L ammonia hydroxide in water (pH = 9.75) and (B) acetonitrile. The injection volume was 2 μL. The mass spectrometer, controlled by Xcalibur software (Thermo Scientific), acquired full-scan MS spectra. ESI source conditions were as follows: capillary temperature 320 ℃; full MS resolution, 60000; spray voltage, 3.8 kV (positive) or -3.4 kV (negative). Raw data were converted to mzXML format using ProteoWizard and processed with an in-house R-based program (built upon XCMS) for peak detection, extraction, alignment, and integration. Metabolite identification utilized the R package and BiotreeDB (v3.0). All identified metabolites underwent orthogonal partial least squares discriminant analysis (OPLS-DA), with metabolites exhibiting a variable importance in project p-value < 0.05 and fold change (FC) ≥ 2 or ≤ 0.5 considered significant.

**Targeted analysis of anthocyanins in mature black rice seeds**

Quantification of anthocyanins in mature black rice seeds was performed using a targeted metabolomics approach. Pretreatment of whole-grain and bran samples followed the procedure used for non-targeted metabolomic analysis, with extraction carried out using 80% methanol (pH = 1.0).

Liquid chromatography-tandem mass spectrometry (LC-MS/MS) was carried out on an Agilent 1290 III UHPLC system coupled to an Agilent 6495D triple quadrupole mass spectrometer. Separation was achieved on a Poroshell 120 EC-C18 column (2.1 × 150 mm, 2.7 µm; Agilent Technologies) with a mobile phase consisting of water containing 0.04% acetic acid and acetonitrile containing 0.04% acetic acid. The injection volume was 5 µL.

Mass spectrometric detection was performed in positive ionization mode with the following parameters: drying gas temperature, 250 °C; drying gas flow, 11 L/min; nebulizer pressure, 35 psi; sheath gas temperature, 350 °C; sheath gas flow, 13 L/min; and capillary voltage, 3 kV. Multiple reaction monitoring (MRM) transitions for each anthocyanin are provided in Table S11.

Data were processed using MassHunter Quantitative Analysis software (version 10.1, Agilent Technologies). The following authentic standards were used for quantification: cyanidin 3-O-glucoside, peonidin 3-O-glucoside, cyanidin 3-O-rutinoside, malvidin 3-O-glucoside, delphinidin 3-O-glucoside, cyanidin 3,5-di-O-glucoside, and peonidin 3,5-di-O-glucoside (EXTRASYNTHESE, Genay, France).

**Construction of co-expression network among metabolites, flavonoid biosynthesis pathway genes, and transcription factors**

We constructed association networks integrating metabolites, biosynthesis genes, and transcription factors (including literature-reported candidates) screened through metabolomic, transcriptomic, and genomic approaches. Pearson correlation coefficients (*r*) between these components were calculated, with pairs showing ∣*r*∣>0.3 connected to establish network edges. The network was visualized and analyzed using Gephi (version 0.10) [18].

**Genome-wide association study**

Only SNPs with minor allele frequency ≥0.05 were used for GWAS. Population structure was modeled as a fixed effect in FaST-LMM using the PCA matrix. We performed GWAS using factored spectrally transformed linear mixed models provided by the FaST-LMM program [19]. The genome-wide significance threshold, calculated at a nominal α = 0.05 with Bonferroni correction, was *p* = 1.23 × 10^−7^ for the mixed population and *p* = 8.94 × 10^−8^ for the black rice population.

**Transport activity assay of OsTT12**

The 1,617 bp full length *OsTT12* cDNA was amplified from the total RNA of pericarp tissues of the black rice variety Huamoxiang No.5 using the primers listed in Table S12, and were then constructed into the yeast expression vector pESC-His. The recombinant expression vector harboring *OsTT12* was transformed into *Saccharomyces cerevisiae* strain WAT 11 for heterologous expression. All procedures including yeast culture, transformation, microsomal fraction preparation, and transport activity assays were performed according to Zhao et al (2009) [20]. For the transport assay, the reaction system (800 μL total volume) contained: 25 mM Tris MES (pH = 8.0), 0.4 M sorbol, 50 mM KCl, 5 mM Mg-ATP, 0.1% (w/v) BSA, and 2 mM substrates C3G or cyanidin. The reaction was initiated by adding 200 μL microsomal suspension and terminated by immediate ice bath incubation. A negative control was prepared identically but without Mg-ATP. After terminating the reaction by ice incubation, the mixture was filtered through 0.22 μm nylon membrane. The membrane was washed six times with 1 mL ice-cold wash buffer (25 mM Tris MES, pH 8.0, containing 0.4 M sorbol). Final elution was performed with 500 μL lysis buffer (80% methanol, pH 2.0, acidified with HCl) to release transported substrates. The eluate was collected for LC-MS analysis. All filtration and washing steps were conducted under positive pressure. All measurements were performed in three independently replicates.

**Yeast-two-hybrid screening**

Yeast AH109 cells were co-transformed with specific bait and prey constructs through the LiCl-PEG method according to the manufacturer’s manual (Clontech, CA, USA). The transformants were selected on SD/-Leu/-Trp medium. The interactions were tested on SD/-Leu/-Trp/-His/-Ade medium.

**Firefly** **luciferase complementation imaging assay**

The luciferase complementation imaging (LCI) assay was conducted as per the protocol described by Chen et al. (2008) [21]. In brief, *Agrobacterium* cells carrying the OsMYB3-nLUC and OsWRKY1-cLUC or OsBBX13-cLUC plasmids were co-infiltrated into *Nicotiana benthamiana* leaves with corresponding empty vectors as a negative control. At 48 h post-infiltration, the infiltrated leaves were gently injected with luciferin (1 mmol/L) and incubated in dark for 5 min before imaging. LUC activities were detected using the Tanon-5200 Chemiluminescent Imaging System (Tanon Science and Technology).

**Subcellular localization assays**

The 1,220 bp and 1,605 bp full-length cDNA of *OsBBX13* and *OsWRKY1* genes were amplified from the total RNA of immature seeds of the black rice variety Huamoxiang No.5 using the primers listed in Table S12 and then constructed into the pM999-mCherry vector through homologous recombination. The recombinant gene-pM999-mCherry plasmid was transformed into rice protoplasts for transient expression. Images were captured using a confocal fluorescence microscope (Olympus FV1200). Cyan fluorescence from the CFP channel, red fluorescence from the mCherry channel and chloroplast fluorescence were observed at the excitation wavelengths of 405, 559 and 488 nm, respectively, and the fluorescence acquisition wavelengths of 460-500, 570-618 and 655-755 nm, respectively.

**Transcriptional activity assay using rice protoplasts**

The cDNAs of the *OsMYB3*, *OsKala4*, *OsTTG1*, *OsWRKY1* and *OsBBX13* genes isolated from Huamoxiang No.5 were inserted into the ‘None’ vector as effectors. Approximately 2-kb promoter regions of the *OsCHS*, *OsCHI*, *OsF3′H*, *OsF3H*, *OsDFR* and *OsANS* genes isolated from Huamoxiang No.5 were inserted into the “190Fluc” vector to drive firefly luciferase (fLUC) as reporters. The internal control vector contains renilla luciferase (rLUC) driven by the *Arabidopsis* ubiquitin promoter. Isolation of rice protoplasts and dual luciferase transcriptional activity assays were performed as described previously [22]. Luciferase activity was measured using the Dual-Luciferase® Reporter Assay System (Promega, Madison, USA). Three independent transformations and measurements for each sample were performed.

**Construction of CRISPR/Cas9 knockout and overexpression vector and genetic transformation**

The construction of CRISPR/Cas9 knockout vectors was performed according to the method described by Ma et al. (2015) [23]. The target sites for gene knockout were designed using the CRISPR design tool available at http://rice.hzau.edu.cn/cgi-bin/rice_rs3/CRISPR_rice. Meanwhile, the full-length cDNA sequences of *OsWRK1* (1,605 bp), *OsBBX13* (1,220 bp), and *OsTT12* (1,617 bp) were isolated from the black rice variety Huamoxiang No.5 and cloned into the plant expression vector pCAMBIA1300 under the control of the maize ubiquitin promoter and NOS terminator to generate overexpression constructs. Subsequently, *Agrobacterium*-mediated genetic transformation of the black rice variety W110 was carried out based on the protocol established by Lin et al. (2002) [24]. Mutation sites and knockout lines were identified in T0 generation transgenic plants through PCR amplification followed by sequencing analysis. Target gene expression of overexpression lines was detected through RT-qPCR in developing seeds at 10 DAF. All primers used in this study were listed in Table S12.

**Development and agronomic comparison of black and white rice sister lines**

Two pairs of black and white rice sister lines were developed from two independent crosses: Huanghuazhan (white rice)/Jinzi No.1 (black rice) and Huamoxiang (black rice)/Jiayouguojing (white rice) as shown in Fig. S13a. The F1 progeny from each cross were self-pollinated to F5 generation, with segregation for pericarp color monitored at each generation. From a single heterozygous F5 line, black and white rice sister lines were isolated in F6 line. Genome DNA of these sister lines was subjected to GreenFaFa Co., Ltd. (Wuhan, China) company for genetic similarity analysis using a 40K SNP array. Genetic similarity (%) was calculated using the following formula: Genetic Similarity = [(Total number of loci - Number of differing loci) / Total number of loci] × 100%.

The two pairs of sister lines were grown at the experimental farm of Huazhong Agricultural University in Ezhou, Hubei province, using a randomized complete block design. Each line was grown in three replicate plots, with 40 plants per plot. Key agronomic traits including plant height, panicle number per plant, grain length, grain width, 1000-grain weight, seed setting rate, and yield per plant were examined at maturity.

**Reference**

1. Doyle JJ, Doyle JL. A rapid DNA isolation procedure for small quantities of fresh leaf tissue. *Phytochem Bull*. 1987; **119**: 11–15. 10.2307/4119796

2. Chen S, Zhou Y, Chen Y *et al.* fastp: an ultra-fast all-in-one FASTQ preprocessor. *Bioinformatics*. 2018; **34**(17): i884–i890. 10.1093/bioinformatics/bty560

3. Li H, Durbin R. Fast and accurate short read alignment with Burrows-Wheeler transform. *Bioinformatics*. 2009; **25**(14): 1754–1760. 10.1093/bioinformatics/btp324

4. McKenna A, Hanna M, Banks E *et al.* The genome analysis toolkit: a MapReduce framework for analyzing next-generation DNA sequencing data. *Genome Res*. 2010; **20**(9): 1297–1303. 10.1101/gr.107524.110

5. Wei X, Qiu J, Yong KC *et al.* A quantitative genomics map of rice provides genetic insights and guides breeding. *Nat Genet*. 2021; **53**(2): 243–253. 10.1038/s41588-020-00769-9

6. Purcell S, Neale B, Todd-Brown K *et al.* PLINK: A tool set for whole-genome association and population-based linkage analyses. *Am J Hum Genet*. 2007; **81**(3): 559–575. 10.1086/519795

7. Kumar S, Stecher G, Li M *et al.* MEGA X: molecular evolutionary genetics analysis across computing platforms. *Mol Biol Evol*. 2018; **35**(6): 1547–1549. 10.1093/molbev/msy096

8. Letunic I, Bork P. Interactive Tree of Life (iTOL) v6: recent updates to the phylogenetic tree display and annotation tool. *Nucleic Acids Res*. 2024; **52**(W1): W78–W82. 10.1093/nar/gkae268

9. Alexander DH, Novembre J, Lange K. Fast model-based estimation of ancestry in unrelated individuals. *Genome Res*. 2009; **19**(9): 1655–1664. 10.1101/gr.094052.109

10. Yang JA, Lee SH, Goddard ME *et al.* GCTA: A tool for genome-wide complex trait analysis. *Am J Hum Genet*. 2011; **88**(1): 76–82. 10.1016/j.ajhg.2010.11.011

11. Chen H, Patterson N, Reich D. Population differentiation as a test for selective sweeps. *Genome Res*. 2010; **20**(3): 393–402. 10.1101/gr.100545.109

12. Danecek P, Auton A, Abecasis G *et al.* The variant call format and VCFtools. *Bioinformatics*. 2011; **27**(15): 2156–2158. 10.1093/bioinformatics/btr330

13. Browning BL, Zhou Y, Browning SR. A one-penny imputed genome from next-generation reference panels. *Am J Hum Genet*. 2018; **103**(3): 338–348. 10.1016/j.ajhg.2018.07.015

14. Kim D, Langmead B, Salzberg SL. HISAT: a fast spliced aligner with low memory requirements. *Nat Methods*. 2015; **12**(4): 357–360. 10.1038/nmeth.3317

15. Pertea M, Pertea GM, Antonescu CM *et al.* StringTie enables improved reconstruction of a transcriptome from RNA-seq reads. *Nat Biotechnol*. 2015; **33**(3): 290–295. 10.1038/nbt.3122

16. Love MI, Huber W, Anders S. Moderated estimation of fold change and dispersion for RNA-seq data with DESeq2. *Genome Biol*. 2014; **15**(12): 550. 10.1186/s13059-014-0550-8

17. Xu SB, Hu ER, Cai YT *et al.* Using clusterProfiler to characterize multiomics data. *Nat Protoc*. 2024; **19**(11): 3292–3320. 10.1038/s41596-024-01020-z

18. Bastian M, Heymann S, Jacomy M. Gephi: an open source software for exploring and manipulating networks. In: *Proceedings of the international AAAI conference on web and social media,* *2009*. Abstract 1, p. 361–362.

19. Lippert C, Listgarten J, Liu Y *et al.* FaST linear mixed models for genome-wide association studies. *Nat Methods*. 2011; **8**(10): 833–835. 10.1038/Nmeth.1681

20. Zhao J, Dixon RA. MATE transporters facilitate vacuolar uptake of epicatechin 3′-O-glucoside for proanthocyanidin biosynthesis in *Medicago truncatula* and *Arabidopsis*. *Plant Cell*. 2009; **21**(8): 2323–2340. 10.1105/tpc.109.067819

21. Chen H, Zou Y, Shang Y et al. Firefly luciferase complementation imaging assay for protein-protein interactions in plants. *Plant Physiol*. 2008; **146**(2): 368-376. 10.1104/pp.107.111740

22. Zong W, Tang N, Yang J *et al.* Feedback regulation of ABA signaling and biosynthesis by a bZIP transcription factor targets drought-resistance-related genes. *Plant Physiol*. 2016; **171**(4): 2810–2825. 10.1104/pp.16.00469

23. Ma X, Zhang Q, Zhu Q *et al.* A robust CRISPR/Cas9 system for convenient, high-efficiency multiplex genome editing in monocot and dicot plants. *Mol Plant*. 2015; **8**(8): 1274–1284. 10.1016/j.molp.2015.04.007

24. Lin Y, Chen H, Cao Y *et al.* Establishment of high-efficiency *Agrobacterium*-mediated genetic transformation system of Mudanjiang 8. *Acta Agronomica Sinica*. 2002; **28**(3): 294–300.
